# Palaeohistology and life history of the early Palaeocene taeniodont *Conoryctes comma* (Mammalia: Eutheria)

**DOI:** 10.1111/joa.70010

**Published:** 2025-07-14

**Authors:** Gregory F. Funston, Zoi Kynigopoulou, Thomas E. Williamson, Stephen L. Brusatte

**Affiliations:** ^1^ School of GeoSciences University of Edinburgh Edinburgh UK; ^2^ Royal Ontario Museum Toronto Ontario Canada; ^3^ Department of Earth and Planetary Sciences University of California, Davis Davis California USA; ^4^ Department of Anatomical Sciences Stony Brook University Stony Brook New York USA; ^5^ New Mexico Museum of Natural History and Science Albuquerque New Mexico USA

**Keywords:** Cimolesta, life history, mammalia, Palaeocene, palaeohistology

## Abstract

The life histories of Palaeocene mammals are poorly known, but may have been central to their success in diversifying across terrestrial ecosystems after the end‐Cretaceous extinction. Among these mammalian groups, the eutherian Taeniodonta are particularly enigmatic, with few modern analogues and no living descendants, despite being one of the only lineages to apparently traverse the Cretaceous‐Palaeogene (K‐Pg) boundary. Here, we investigate the life history of an early Palaeocene taeniodont, *Conoryctes comma*, based on a multi‐individual, multi‐element sample. Nearly all elements sampled exhibit similar osteohistological architecture, with a small internal zone of compacted coarse cancellous bone surrounded by an internal cortex of periosteally derived fibrolamellar bone of variable thickness, and an outer cortex of lamellar bone. The well‐vascularized fibrolamellar complex in the limb bones, lacking cyclical growth marks, is indicative of overall rapid growth to near adult body size. Cyclical growth marks are present in the outer cortex after the transition to slow‐growing lamellar bone, but not in the inner cortex, suggesting sexual maturity was reached in 1 year. In some elements, an internal non‐cyclical growth mark shares histological similarities with weaning marks in living mammals and other contemporary Palaeocene mammals, and occurred at the body size predicted for this transition in therian mammals. The unusual presence of compacted coarse cancellous bone near the midshafts of multiple limb bones may be related to cortical thickening, and is similar to the arrangement described in some fossorial mammals, supporting previous assertions of this lifestyle in *Conoryctes*. Altogether, these palaeohistological signals suggest a life history in *C. comma* similar to living eutherians, despite uncertainty about whether it is within crown Placentalia or a close outgroup. Thus, our data are consistent with an early origin of placental‐like reproductive strategies in their eutherian ancestors, although this attribute was likely shared more broadly among Mesozoic mammal lineages prior to the end‐Cretaceous extinction.

## Graphical abstract

Determining the life histories of mammals after the extinction of the dinosaurs is key to understanding why they rose so quickly to terrestrial dominance. This study analyses growth in a member of a key mammal group that traversed the extinction boundary. The results show that this ancient mammal had growth rates, weaning masses and lifespans relatively similar to living placental mammals of its size, reinforcing the idea that these strategies contributed to the rise of placental mammals.

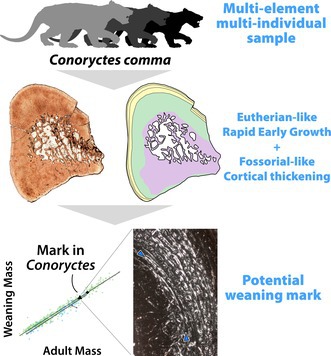

## 1 INTRODUCTION

The transition from dinosaur‐ to mammal‐dominated ecosystems at the beginning of the Palaeocene (66 ma) marked a major rearrangement of terrestrial ecosystems (Alroy, 1999; Grossnickle et al., 2019; Simpson, 1937). The Cretaceous‐Palaeogene (K‐Pg) extinction of non‐avian dinosaurs and Mesozoic mammaliaforms sparked an adaptive radiation of so‐called ‘archaic’ eutherian mammals, which nevertheless exhibited a wide range of body sizes, sensory adaptations and ecological niches (Bertrand et al., 2022; Brocklehurst et al., 2021; Chester et al., 2017; Shelley et al., 2021; Williamson, 1996). These eutherians were predominant in their ecosystems, at least in North America, but were joined by a relatively smaller cast of multituberculates and metatherians, which had been the eminent mammal groups prior to the extinction (Lyson et al., 2019; Wilson, 2013, 2014; Wilson et al., 2012, 2016). Thus, these Palaeocene ecosystems exhibited differences from Cretaceous faunas not only in their overall architecture, but also within the components of the mammal assemblages.

Based on living members of these major clades, an influential hypothesis has arisen that these differences in mammalian ecological success in the early Palaeocene reflected aspects of their life histories, specifically their reproductive cycle and maternal investment strategies (Lillegraven, 1975; Lillegraven et al., 1987). However, contrasting evidence from other groups suggests that the eutherian strategy may not have been unique in the early Palaeocene (Weaver et al., 2022). Problematically, little is known of the reproductive strategies or broader life histories of most ‘archaic’ eutherians (Funston et al., 2022), which makes it difficult to resolve this dilemma. Therefore, addressing this hypothesis robustly requires detailed data about the growth and pace of life in ‘archaic’ eutherians and their contemporaries.

Among ‘archaic’ eutherians, Taeniodonta are enigmatic but particularly salient to discussions of eutherian growth. They are one of the few mammal clades that has been proposed to have traversed the Mesozoic–Cenozoic boundary, based on inferred relationships with the Late Cretaceous *Schowalteria* (Fox & Naylor, 2003) and possibly among other ‘cimolestids’, although their phylogenetic positions are debated (Fox, 2016; Kynigopoulou, 2023; Rook & Hunter, 2011, 2013; Velazco et al., 2022). After the extinction, they are represented by a spate of medium‐ to large‐sized Palaeocene and Eocene species (Rook & Hunter, 2011; Williamson & Brusatte, 2013). Nine genera and two families of taeniodonts are recognized from the Cenozoic, which all share high degrees of dental wear (Kynigopoulou, 2023; Schoch, 1981). Despite a long history of study (Cope, 1876), postcranial skeletons of the group are relatively rare, although those known exhibit adaptations for scratch‐digging (Kynigopoulou, 2023; Kynigopoulou et al., 2024). Altogether, their highly worn teeth and their patchy postcranial fossil record mean that the phylogenetic position of taeniodonts is debated, and they are either regarded as early‐diverging placentals, or as non‐placental eutherians just outside of the crown group (Halliday et al., 2017; Rook & Hunter, 2013; Shelley and Paleocene Mammal Working Group, 2022). Therefore, their growth rates and life histories may bear directly on hypotheses of differential survivorship at the K‐Pg boundary and the rise of placental mammals afterwards.

Palaeohistology, the study of fossilized tissues (particularly skeletal tissues in vertebrates), offers an avenue to investigate life‐history parameters in extinct organisms. Numerous studies have shown that the textures of skeletal tissues reflect the rates at which they were deposited (Amprino, 1947; Castanet et al., 2000; de Margerie, 2004; de Margerie et al., 2002; Montes et al., 2007) and that cyclical marks within the skeleton can be attributed to periodic events, like daily cycles or annual seasons (Castanet, 2006; Castanet et al., 1990, 2004; de Buffrénil et al., 2021; Emken et al., 2021; Kierdorf et al., 2013; Klevezal, 1996; Köhler et al., 2012; Padian & Lamm, 2013). Altogether, these signals allow for qualitative (and/or more rarely, quantitative) estimation of growth rates, life spans and life histories (Chinsamy, 1990, 1994; Chinsamy & Elzanowski, 2001; Chinsamy & Hurum, 2006; Cullen et al., 2020; Erickson, 2000; Erickson et al., 2001, 2004, 2007, 2017; Funston et al., 2022; Köhler et al., 2023; Köhler & Moya‐Sola, 2009; Kolb et al., 2015; Montoya‐Sanhueza et al., 2019; Montoya‐Sanhueza & Chinsamy, 2017; Padian & de Ricqlès, 2020).

With regard to taeniodonts, nothing is known of their palaeohistology, and some peculiar aspects of their palaeobiology make their growth more difficult to assess than other mammal clades: Excessive accumulation of dental wear prohibits access to the daily records of growth normally chronicled in enamel and dentine (Dean, 2006, 2017; Dirks et al., 2009; Dirks & Bowman, 2007; Emken et al., 2021; Funston et al., 2022; Kierdorf et al., 2013, 2019; Klevezal, 1996; Smith, 2006, 2013). Instead, the current fossil record of taeniodonts means that investigation of their life histories must rely predominantly on bone histology.

Here, we provide a first step in addressing this knowledge gap by documenting the palaeohistology of the unusual eutherian group Taeniodonta for the first time. We perform multi‐individual, multi‐element palaeohistological analysis on *Conoryctes comma*, a medium‐sized taeniodont from the Torrejonian North American Land Mammal Age (NALMA) of the San Juan Basin, New Mexico. The objective of this study was not to test any particular hypotheses about the growth of *C. comma*, as no preliminary data exist to formulate them, but instead to provide an initial description and comparison of the osteohistology of this taxon and to generate hypotheses about its life history and growth strategy. The results provide the first information about growth and life history in a taeniodont mammal, with further ramifications for the diversity of life histories in eutherian mammals in the early Palaeocene.

## 2 MATERIALS AND METHODS

### 2.1 Specimens

**FIGURE 1:**
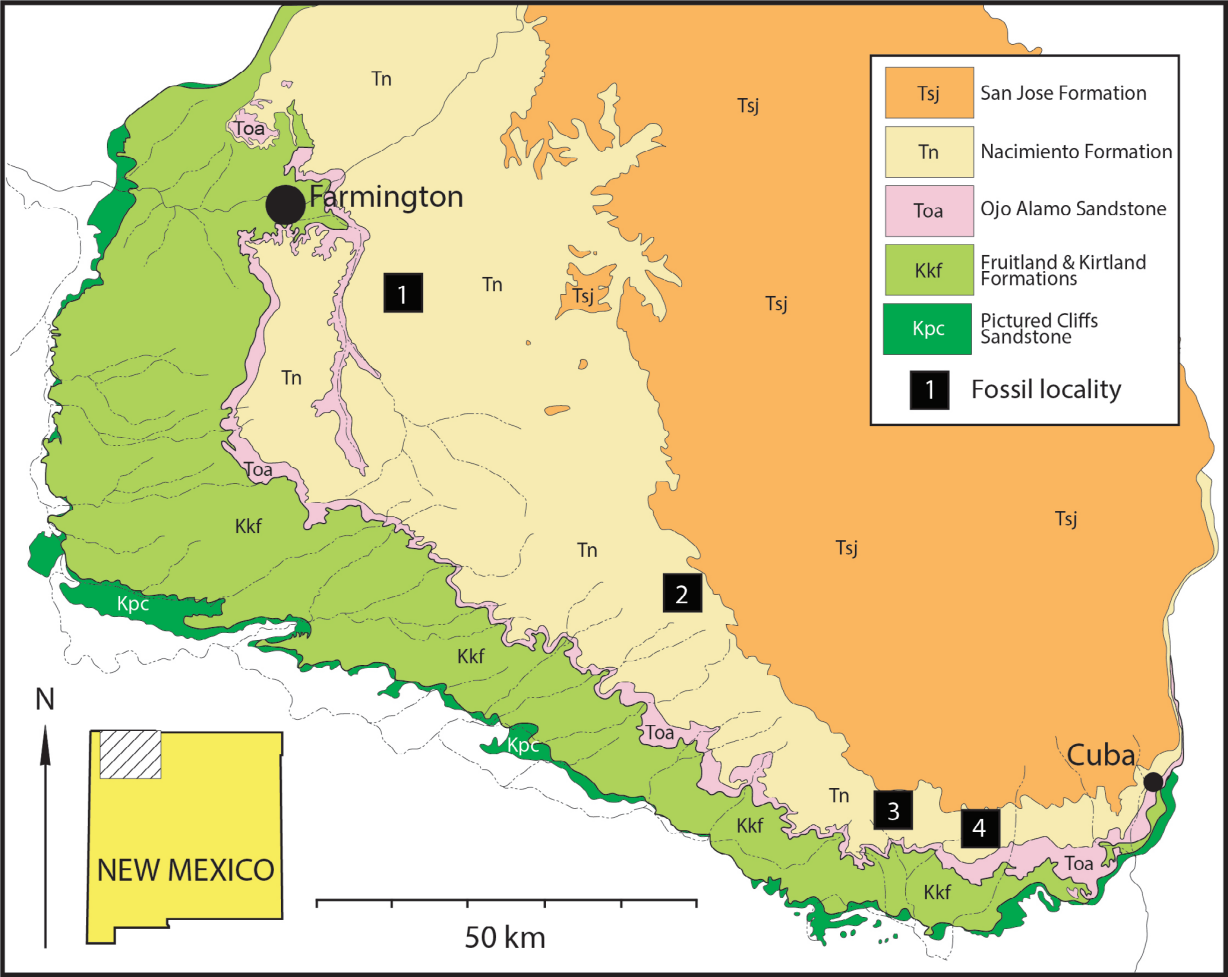
Geologic map of the San Juan Basin, New Mexico showing Upper Cretaceous though lower Eocene strata. Specimens of *Conoryctes comma* were collected from the lower Palaeocene of the Nacimiento Formation. Fossil localities are: (1) Kutz Canyon (NMMNH L‐6419; middle Torrejonian [To2]); (2) Red Mesa, Head of Kimbeto Wash (NMMNH L‐10992; To2); (3) West Flank Torreon Wash (NMMNH L‐1287, 6251, 6286; late Torrejonian [To3]); (4) East Flank Torreon Wash (NMMNH L‐2687; To3).

We selected a range of postcranial skeletons of *C. comma*, all collected from the Nacimientio Formation in the San Juan Basin of New Mexico (Figure 1). In this paper, we consider *Conoryctes* to likely be synonymous with, or very closely related to, another named taeniodont genus, *Huerfanodon*, following Kynigopoulou (2023). Both taxa overlap in time and are nearly identical in size, being distinguished only by relatively minor differences in premolar morphology (e.g. presence/absence of P4 protocone; presence/absence of p4 metaconid), which is highly variable even between members of the same species (Kynigopoulou, 2023). However, it is outside the scope of this study to formally synonymize these names, and this endeavour is in progress elsewhere. Where the postcranial material in our sample is associated with dental material (e.g. NMMNH P‐48198), the teeth are referable to *C. comma* (Kynigopoulou et al., 2024). Further, because duplicate postcranial elements are identical in morphology and palaeohistological growth signals (i.e. they do not exhibit different growth trajectories), we consider them all to represent a single taxon, *C. comma*. The San Juan Basin where the specimens were collected is the historic territory of the Ute and Navajo (Diné) peoples, and all specimens were collected under permit from public lands administered by the United States Bureau of Land Management. All specimens are accessioned at the New Mexico Museum of Natural History and Science (NMMNH). Specimens range in age from middle (To2) to late (To3) Torrejonian, spanning from about 62.7 to 62.2 Ma (Flynn et al., 2020; Leslie et al., 2018). Specimen details and parts of the specimens sampled are outlined in Table 1.

**TABLE 1:** Sampled specimens and elements of *Conoryctes comma*.

Specimen number	Locality	Elements sampled	Total thin sections
NMMNH P‐19494	L‐1287	Tibia	1
		Limb fragment	1
NMMNH P‐21509	L‐2687	Tooth root	1
		Tibia	3
NMMNH P‐47700	L‐6419	Humerus	2
		Tibia	1
		Limb fragment	1
		Bone fragments	2
NMMNH P‐48052	L‐6251	Humerus	2
		Ulna	2
		Femur	1
		Tibia	2
		Limb fragment	1
		Autopodial bone	3
		Ribs	2
		Bone fragments	4
NMMNH P‐48198	L‐6286	Tibia	2
NMMNH P‐79457	L‐10992	Humerus	1
		Fibula	1
		Radius	3
Total			36

The sample aimed to include not only multiple individuals of varying sizes, for evaluating ontogenetic variation in tissue types, but also multiple elements from each individual, to assess intraskeletal histovariability. This is because bones from individuals of different ages record different windows of life and bones from different regions of the skeleton can vary in how their local growth patterns reflect overall mass increase in the body. Thus, broad sampling is necessary when studying lineages with poor histological characterization, particularly because the elements whose growth records most closely reflect overall body mass increase are not necessarily consistent between clades (Cullen et al., 2014; Horner et al., 2000; Klevezal, 1996; Kolb et al., 2015; Montoya‐Sanhueza et al., 2021; Padian et al., 2013, 2016; Woodward, 2019; Woodward et al., 2014, 2015). In *Conoryctes*, like in other Palaeocene eutherians studied (Funston et al., 2022), we found minimal variation in the overall growth signal between bones (see discussion). Where possible, sampling targeted the minimum shaft circumference of each bone, because this is typically closest to the neutral growth zone and more closely reflects the overall growth of the individual (Enlow, 1963; Padian et al., 2013), particularly in simple cylindrical elements like the tibia. However, in many cases, this area was not preserved; the bones had more complex shapes; or the specimens available for sampling were small but identifiable fragments of the element. Accordingly, some sections had to be taken closer to the metaphyses, which typically have more complex growth signals that reflect not only size increase, but also shape change and soft tissue attachments. Likewise, for some sections, only an approximate position of the sample relative to the entire element is known.

### 2.2 Methods

Specimens were photographed using a Nikon D850 camera with a 60 mm MicroNikkor lens prior to thin sectioning. Some elements were digitized using photogrammetry in Agisoft Metashape Standard (version 1.6.1), to produce three‐dimensional models for conservation of morphology.

Specimens were thin sectioned following the protocol of Funston et al. (2022), which is a modified version of the method described by Lamm (2013). In brief, sample regions were removed from specimens either using natural breaks, or by stabilizing with paraloid and cutting on a Buehler Isomet 1000 Precision saw. A major difference from other thin‐sectioning protocols is that sample regions were adhered in predetermined orientations to pre‐cured epoxy bases using Loctite cyanoacrylate, to facilitate sampling in the correct plane. Specimens on bases were then embedded in Buehler Epothin II epoxy resin under a vacuum and left to cure for 24 h. Embedded blocks were sectioned at the plane of interest using the Isomet 1000 saw, but unlike the protocol of Lamm (2013), specimens were kept as blocks, rather than serially cut into wafers, as this minimized the potential for loss of the friable bones and teeth. Cut block faces were consolidated using cyanoacrylate as necessary, before being frosted using 600 grit silicon carbide abrasive slurry. Unlike most thin‐sectioning protocols (Cerda et al., 2020; Chinsamy & Raath, 1992; Cuccu et al., 2025; Lamm, 2013; Montoya‐Sanhueza & Chinsamy, 2017), but critical for optimal adhesion of fragile dental tissues, frosted acrylic slides were inverted to mount onto the blocks using cyanoacrylate without applying any pressure. Typically, glass slides and epoxy adhesive are used, and specimens are weighed down or clamped for mounting; but this often temporarily warps the slide, which results in flexion and breakage of dental tissues when slides are polished to low thicknesses. Cyanoacrylate and acrylic make a stronger bond than glass and epoxy, because the acrylic ‘melts’ in contact with cyanoacrylate, merging into a single material, and thus the bond does not rely on the surface area adhesion of two materials (epoxy and glass). Acrylic provides a further advantage in that it is more resistant to shattering, prolonging the lifespan of the completed section. Mounted samples were resectioned using the Isomet 1000 saw to a thickness of 700 μm, and were then manually ground on a glass plate using 600‐ and 1200‐grit silicon carbide abrasive slurries. Slides were ground until they reached the desired optical clarity, although for many specimens in the sample, taphonomic or diagenetic alteration of the material resulted in replacement of original bone matrices with extensive regions of opaque minerals. Because of the modified mounting protocol, these slides could be polished to very low thicknesses, in some cases, <15–30 μm thick, to maximize light transmission. Thus, some information was still accessible from these specimens using cross‐polarized light, which would not have been available using standard palaeohistological methods.

Slides were imaged using a Leica DMLP Transmitted Light Polarizing Microscope with Leica Application Suite 4. Photomontages were created using the automated ‘photomerge’ feature of Adobe Photoshop 2022. Images were taken under plane polarized light, cross‐polarized light and cross‐polarized light with a 430 nm ‘lambda’ filter, which aids in distinguishing opaque regions from extinct regions under cross‐polarized light. Images of the slides are available at https://doi.org/10.5061/dryad.vhhmgqp6f.

### 2.3 Terminology

Palaeohistological terminology follows Padian and Lamm (2013) and de Buffrénil et al., (2021), which themselves are generally modified from Francillon‐Vieillot (1990) or Enlow (1963). Although some authors recommend the use of the broader ‘woven‐parallel’ complex in place of ‘fibrolamellar’ (Prondvai et al., 2014; Stein & Prondvai, 2014), we prefer the latter term because of its widespread use, and because in its original formulation, ‘woven‐parellel’ was part of a dichotomy with a new definition for ‘parallel‐fibred’ bone. ‘Parallel‐fibred’ bone was originally used to refer to tissues which show distinctive semi‐aligned collagen orientations in transverse section, appearing intermediate between woven and lamellar tissues (Francillon‐Vieillot, 1990; Huttenlocker et al., 2013). However, Stein and Prondvai (2014) redefined ‘parallel‐fibred’ bone as an umbrella term including all highly organized tissues derived from dynamic osteogenesis, whether lamellated or not, equivalent to what most authors would previously call lamellar tissues. We prefer to use the term ‘parallel‐fibred’ as defined by Francillon‐Vieillot (1990) and Huttenlocker et al. (2013). This is because, in our samples, there is more continuity between the parallel‐fibred tissues (sensu Huttenlocker et al., 2013) and the fibrolamellar tissues than between the former and lamellated highly organized tissues. Furthermore, in our samples, the parallel‐fibred tissues appear intermediate in organization between the two other categories. Because we find this variation important and noteworthy in our study, we prefer to describe them as parallel‐fibred tissues under the original definition, and so using ‘woven‐parallel’ to describe fibrolamellar tissues would be misleading.

Altogether, we delineate three primary tissue complexes in our sample, representing a gradient of progressively greater collagen fibre orientation: (1) Fibrolamellar complex: A mix of tissues, formed of woven bone with large globular osteocyte lacunae infilled with lamellar bone with smaller, lenticular osteocyte lacunae. (2) Parallel‐fibred bone: An intermediate tissue which shows greater organization and anisotropy, and which may gradually transition from the woven component in a fibrolamellar complex. (3) Lamellar bone: Tissues with no discernible woven component and only small, lenticular osteocyte lacunae; equivalent to Prondvai et al. (2014)'s lamellated highly organized primary bone.

Where possible, descriptions aim to provide information on the bone matrix, bone cells, vasculature, growth marks, secondary remodelling and other salient features. However, in many cases, one or more of these categories could not be observed because of diagenetic alteration of the material. This alteration consists of the replacement of normally translucent bone material with opaque minerals, which obscure histological features. Most samples show some degree of alteration that prevents characterization of the bone matrix, bone cells and in many cases, the vasculature, in part or all of the section. Throughout the sample, cyclical bright lines can sometimes be discerned in parts of the cortices that have undergone replacement, particularly towards the periosteal surface. These lines are oriented parallel to the periosteal surfaces, and in some specimens, they can be traced to unambiguous cyclical growth marks (e.g. lines of arrested growth) in adjacent unaltered tissues. Therefore, we interpret these diagenetically altered bright lines as cyclical growth marks representing annual periods (Castanet, 2006; Castanet et al., 1990, 2004; Köhler et al., 2012).

In most of our specimens, there is a prominent transition within the cortical bone from a well‐vascularized fibrolamellar complex internally to a zone with lamellar matrix and reduced vascularity. For ease of reference, we mark the boundary between the ‘inner’ and ‘outer’ cortex at this transition, rather than using arbitrary proportions (e.g. ½, or 1/3 of the radius) of the cortex. Therefore, references to the inner cortex refer to the fibrolamellar complex, and those to the outer cortex refer to the lamellar zone, rather than to fixed proportions of cortical tissue.

Due to the frequency and ubiquity with which we identify compacted coarse cancellous bone (CCCB) in our sample, this tissue type merits a brief mention. CCCB is a mixed tissue complex resulting from endosteal bone deposition infilling trabecular spaces (Enlow, 1962, 1963; Heck et al., 2019; Kolb et al., 2015; Legendre & Botha‐Brink, 2018; Montoya‐Sanhueza & Chinsamy, 2017; Weaver et al., 2022). It is inherently a mix of both primary and secondary tissues, deposited sequentially rather than simultaneously, but this does not necessarily always involve remodelling in the sense that erosion of tissue need not occur. Nevertheless, some degree of secondary remodelling (i.e. erosion of old tissue and deposition of new tissue) is often present in conjunction with CCCB (Enlow, 1963; Montoya‐Sanhueza et al., 2021; Montoya‐Sanhueza & Chinsamy, 2017, 2018). In thin section, CCCB appears disorganized, but with a mix of bone matrix types, ranging from disorganized woven bone to highly organized lamellar bone matrix. Under normal light, it can be initially mistaken for a fibrolamellar complex because it has a mix of woven and lamellar bone matrices, and it often has vasculature, including true primary osteons, which themselves may be part of a fibrolamellar complex. However, it is easily identifiable under cross‐polarized light, where large, bright streaks of endosteal lamellae are oriented haphazardly and often truncate and/or cross cut (de Buffrénil et al., 2021; Enlow, 1963; Legendre & Botha‐Brink, 2018; Montoya‐Sanhueza & Chinsamy, 2017). Furthermore, unlike in fibrolamellar bone, in CCCB, lamellar bone matrices may have formed the initial trabeculae that were compacted, whereas in true fibrolamellar bone, lamellar matrices typically occur as centripetal infill of vascular spaces or in association with other discrete features like growth marks (de Buffrénil et al., 2021).

## 3 RESULTS

Palaeohistological sections show generally mediocre histological preservation, with nearly every element showing some degree of mineral replacement by opaque minerals. Nevertheless, this replacement is rarely complete, and using cross‐polarized light, the overall architecture of the cortical bone is apparent in most specimens. Two specimens show relatively good histological preservation: the tibia of NMMNH P‐48198, wherein the middle cortex shows low abundances of opaque minerals, and NMMNH P‐48052, in which most elements (humerus, tibia, femur, manual/pedal bones, ribs and indeterminate fragments) show excellent histological preservation. Accordingly, these two specimens comprise the bulk of the histological descriptions, with overall cortical arrangements informed by other specimens as appropriate. For brevity and because of similarity between individuals, descriptions are arranged by skeletal element, rather than by individual. Further observations about intraskeletal histovariability are outlined in the discussion section.

### 3.1 Humerus

**FIGURE 2:**
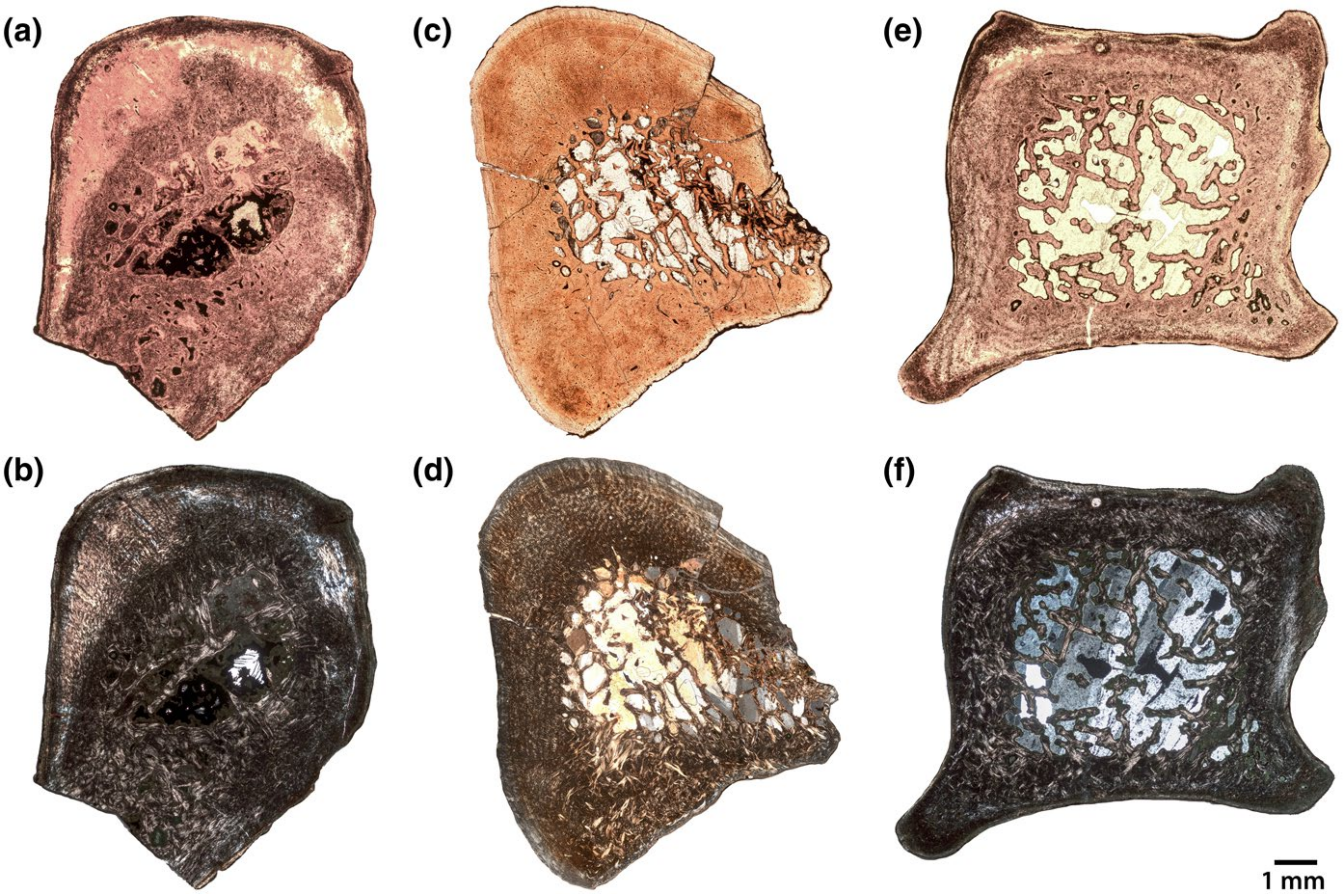
Humeri of *Conoryctes comma*. Thin sections of humeri under plane‐polarized light (a, c, e) and cross‐polarized light (b, d, f) of NMMNH P‐47700 (a, b), P‐48052 (c, d), and P‐79457 (e, f). Images to scale, anterior is to the bottom. The humerus of *C. comma* is incompletely known, making it unclear where exactly along the length of the element the samples were taken.

**FIGURE 3:**
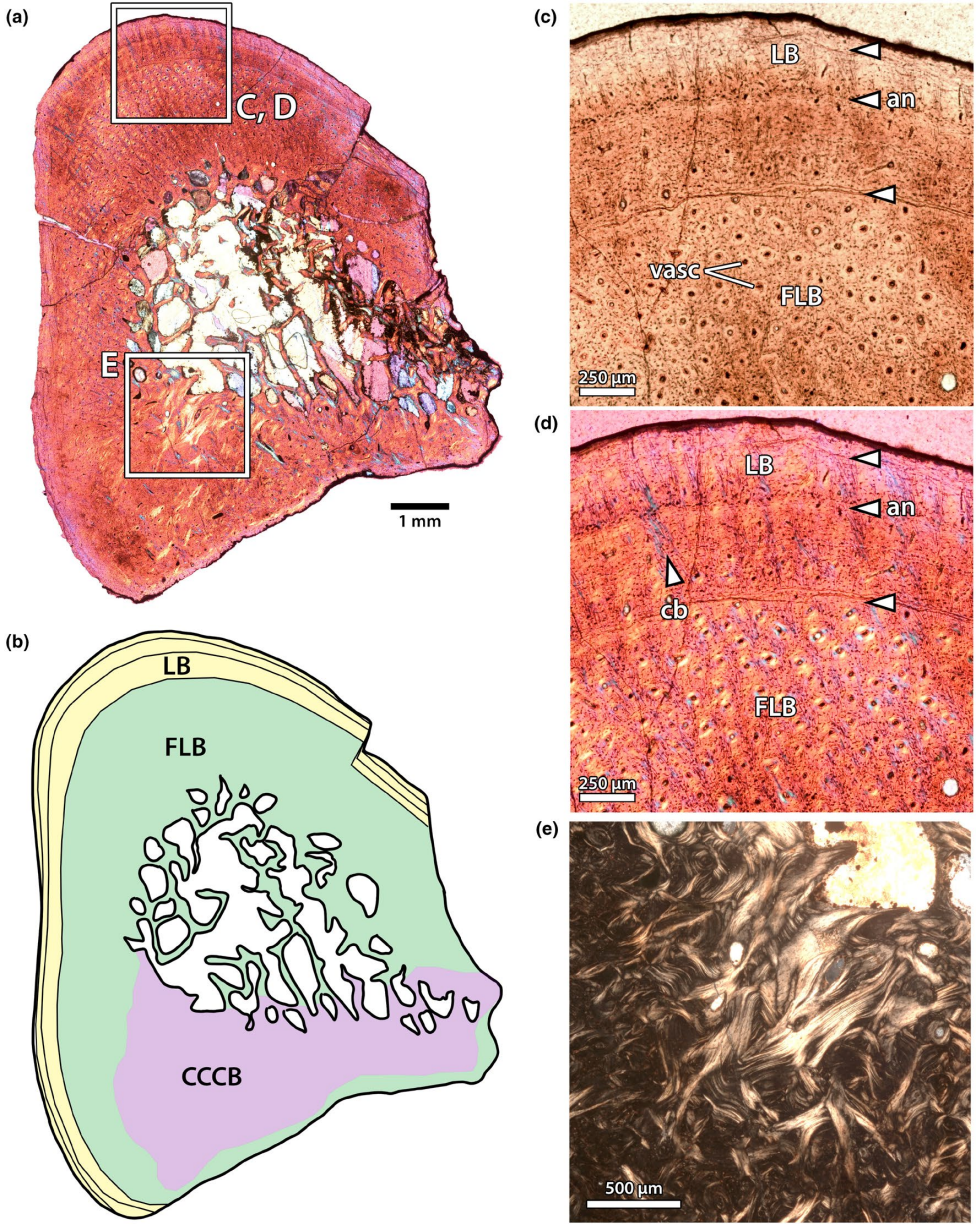
Humerus of NMMNH P‐48052. Thin section under lambda cross‐polarized light (a) and interpretive drawing (b) of the well‐preserved humerus, showing overall architecture and zonation of the cortical bone. Close‐ups (c–e) of the outer cortex in normal (c), lambda cross‐polarized (d) and the compacted coarse cancellous bone in cross‐polarized light (e), showing palaeohistological features including longitudinal vasculature, cyclical growth marks (arrows) and collagen bundles (cb). An, annulus; cb, collagen bundle; CCCB, compacted coarse cancellous bone; FLB, fibrolamellar complex; LB, lamellar bone; vasc, vascular canal. Scale bars as indicated, anterior is to the bottom in (a) and (b), periosteal is to the top in (c–e).

Three humeri were sampled: NMMNH P‐47700, P‐48052 and P‐79457 (Figure 2). The first two specimens were sampled distal to the deltopectoral crest, whereas NMMNH P‐79457 was sampled at the deltopectoral crest. The palaeohistology of the humerus of NMMNH P‐48052 is excellent (Figure 3), with virtually no replacement by opaque minerals, whereas NMMNH P‐47700 (Figure 2a,b) has some areas of replacement, and NMMNH P‐79457 (Figure 2e,f) has extensive replacement, but the overall architecture can still be discerned.

In all specimens, there is a well‐developed medullary cavity with trabecular bone, surrounded by a relatively dense and thick cortex (Figure 2). Adjacent to the medullary cavity, each specimen has zones of endosteally derived compacted coarse cancellous bone (CCCB), external to which is the periosteal cortex (Figure 3b). The inner periosteal cortices are comprised of fibrolamellar bone, with a high proportion of woven bone and primary osteons formed of lamellar bone (Figure 3c). Osteocytes in the woven component are dense and globular, but in the lamellar infilling of the osteons, the osteocytes are more lenticular (although only slightly less dense). The orientations of vasculature in the internal cortical bone vary between the samples. In NMMNH P‐48052, which is sampled closest to the minimum diaphyseal circumference, and in NMMNH P‐79457, the vasculature is longitudinal and relatively dense. In NMMNH P‐47700, there are more radially oriented canals, but the density of vasculature is sparser overall.

In each specimen, there is a transition from the well‐vascularized inner cortex to a thinner external cortex with (a) lower vascularity; (b) an increase in the parallel‐fibred and lamellar components of the bone matrix; and (c) the development of annuli or lines of arrested growth (Figure 3d). This external zone is thicker in NMMNH P‐48052 than in the other specimens, and it is more vascularized with a higher proportion of woven‐fibred matrix (Figure 3d). In this specimen, the transition between the inner and outer cortex is marked by a prominent line of arrested growth (LAG; Figure 3c), and at least one annulus and a further LAG are present within the outer zone. Discerning growth marks in the other specimens is difficult, but NMMNH P‐47700 has three clear bright annuli within the external cortex, whereas NMMNH P‐79457 has one line in addition to the mark that differentiates the inner and external cortices (Figure 2).

All specimens exhibit large bundles of collagen fibres oriented perpendicular to the sub‐periosteal surface, which are especially prominent in the external cortex where there are higher proportions of lamellar matrix (Figure 3d). With the exception of the CCCB near the medullary cavity (Figure 3e), there is little evidence of remodelling in the humeri of *C. comma*.

### 3.2 Radius

**FIGURE 4:**
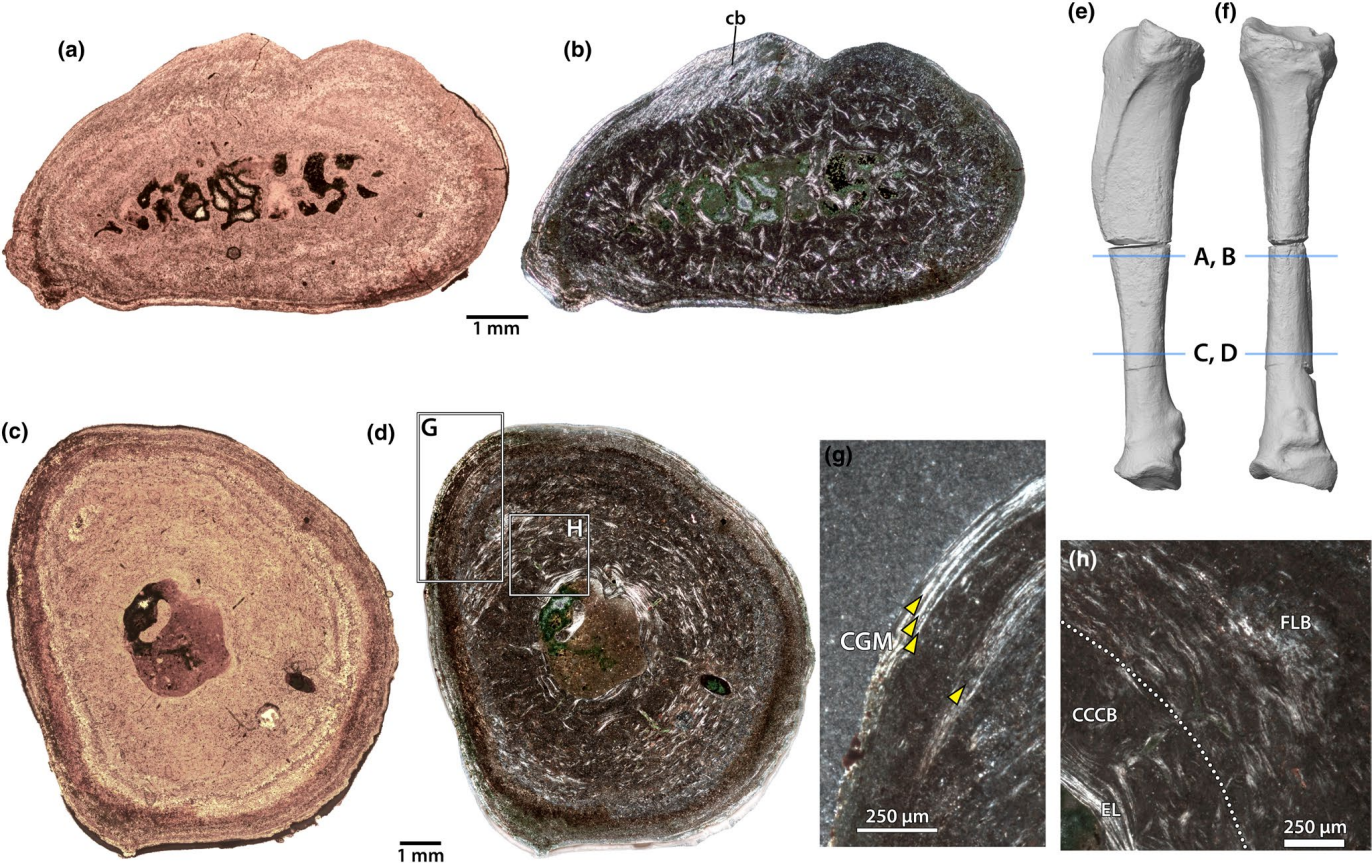
Radius of *Conoryctes comma*. Midshaft (a, b) and proximal (c, d) sections of the radius of NMMNH P‐79457 under normal (a, c), and cross‐polarized light (b, d). Photogrammetric model (e, f) of the radius in lateral (e) and posterior (f) views (distal towards the top), showing the locations of the thin sections. Close up (g) of the outer cortex of the distal section, showing four bright lines interpreted as cyclical growth marks (yellow arrows). Close up (h) of the inner cortex, showing tissue zonation and boundary between CCCB and primary bone. Abbreviations: cb, collagen bundles; CCCB, compacted coarse cancellous bone; CGM, cyclical growth marks; EL, endosteal lamellae; FLB, fibrolamellar bone. Scale bars as indicated.

The radius of NMMNH P‐79457 was sampled in two areas, one towards the distal end of the shaft and another further proximal, at the minimum circumference of the bone (Figure 4). Both regions exhibit mineral replacement, but the overall tissue arrangements can be determined using cross‐polarized light. The medullary cavity is small in the proximal section, whereas it is expanded and has more trabeculae in the distal section (Figure 4a–d). Both sections exhibit areas of CCCB immediately adjacent to the medullary cavity; this area comprises a larger portion of the distal section than the proximal section.

In the distal section, the internal half of the cortex is comprised of primary fibrolamellar bone with sparse longitudinal vascular canals. This is capped externally by a thin rind of lamellar bone with no vascular canals (Figure 4g). This arrangement characterizes nearly the entire circumference of the bone, but there is an area on the medial surface of the distal radius where the transition between these tissue types is much wider and exhibits large, obliquely oriented collagen bundles (Figure 4b). This likely represents the attachment of a soft tissue structure, probably the interosseous membrane, as the external surface of the bone is rugose in this area. At least three bright lines can be discerned in the outer cortex, probably corresponding to annual growth marks, but they cannot be traced fully around the circumference of the bone.

In the proximal section (Figure 4c,d), the internal cortical zone of fibrolamellar tissue comprises more of the cross section of the bone than in the distal section, roughly three quarters of the cortical thickness. This fibrolamellar bone has relatively sparse vascularity, and most of the primary osteons are oriented longitudinally, but some simple vascular canals extend radially, especially closer to the region of CCCB. Unlike the distal section, the medullary cavity in the proximal section has few trabeculae, and its margin is finished with a thick layer of endosteal lamellae. A large foramen, probably the nutrient foramen, pierces the dorsolateral side of the bone. In the outer cortex, which is roughly the same thickness as in the distal section, three bright lines can be traced fully around the circumference of the bone (Figure 4e).

As with the humerus, there are few signs of extensive secondary remodelling of the bone, in either proximal or distal sections, with the exception of CCCB.

### 3.3 Ulna

**FIGURE 5:**
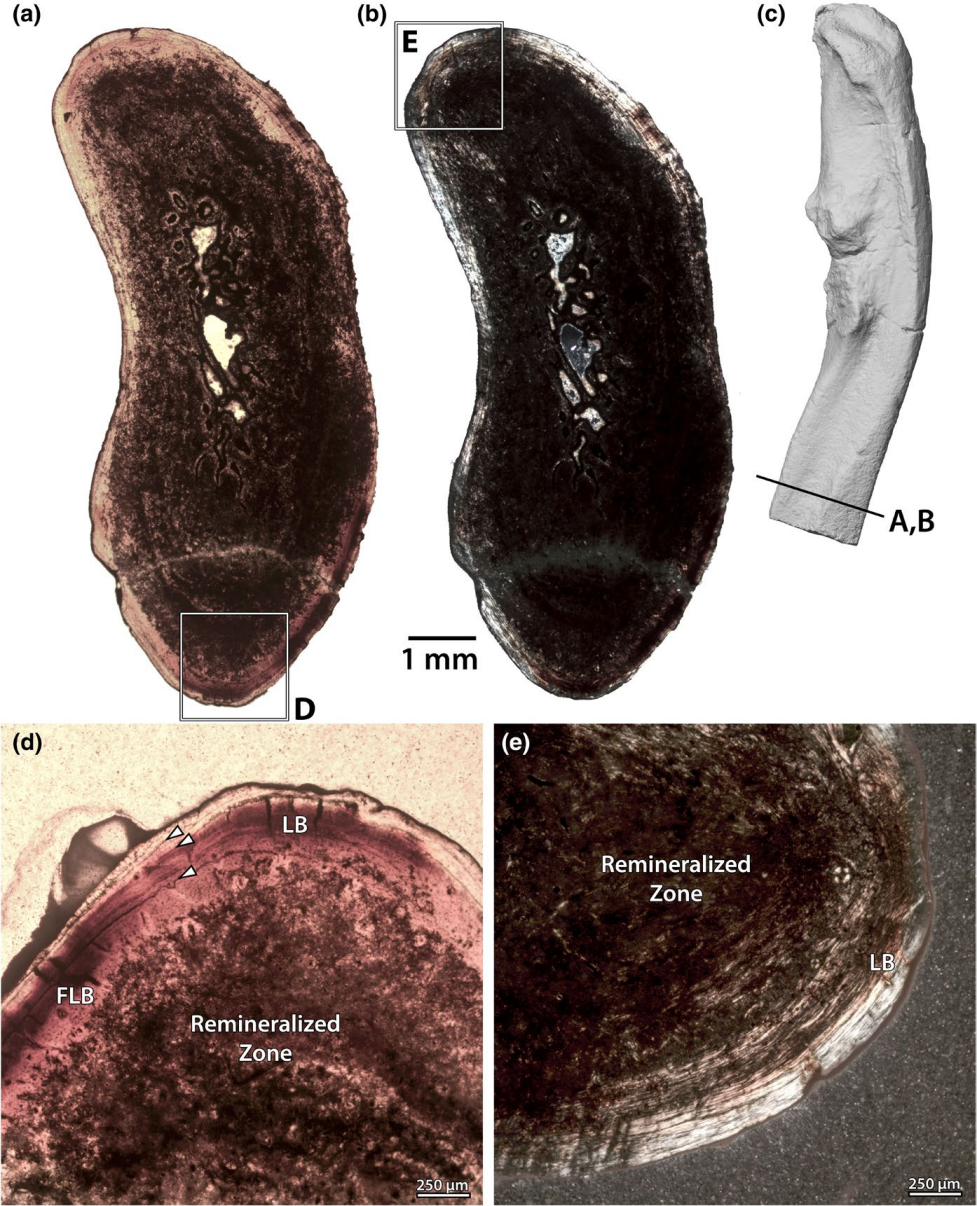
Ulna of *Conoryctes comma*. Ulna of NMMNH P‐48052 in normal (a) and cross‐polarized (b) light, showing extensive remineralization of the bone with opaque minerals. Photogrammetric model (c) of the ulna in medial view (distal towards the bottom), showing the locations of the thin sections. Close ups (d, e) of the outer cortex showing lines of arrested growth (arrows) under normal light (d) and extensive lamellar bone under cross‐polarized light (e). FLB, fibrolamellar bone; LB, lamellar bone. Scale bars as indicated.

The ulna of NMMNH P‐48052 was sampled (Figure 5), but nearly the entire cortex is replaced by especially pervasive opaque minerals. These mostly obscure the inner cortex, its matrix characteristics and the orientation and density of vasculature, but a small rind of unaltered bone is present around the periphery of the section (Figure 5d,e). This reveals fibrolamellar bone with predominantly longitudinal vasculature immediately internal to an external cortical layer of lamellar bone with only a few simple vascular canals. Nevertheless, this suggests that, like other forelimb bones, the cortex of the ulna was formed predominantly of fibrolamellar bone, with a thin rind of lamellar bone at the periosteal surface. Some banding within the opaque minerals suggests that there might also have been an area of CCCB adjacent to the medullary cavity, but this cannot be confirmed with the available thin sections. At least three cyclical growth marks are present in the outer cortex (Figure 5d), and, like the humerus of the same individual, the mark delineating the fibrolamellar and lamellar zones is particularly accentuated.

### 3.4 Femur

**FIGURE 6:**
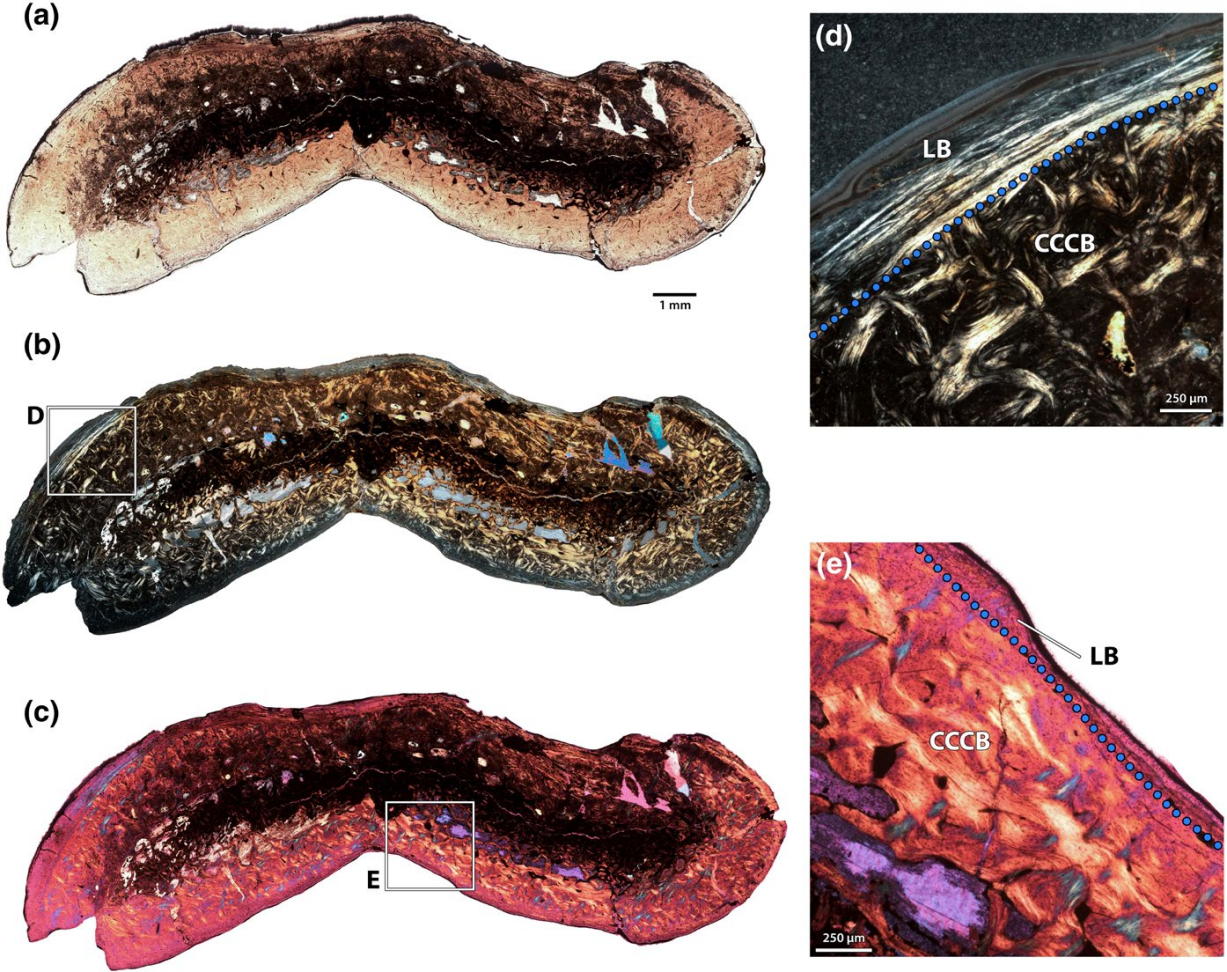
Femur of *Conoryctes comma*. Distal femur of NMMNH P‐48052 under normal (a), cross‐polarized (b), and lambda cross‐polarized (c) light, showing crushing and extensive compacted coarse cancellous bone. Close ups of periosteal surface on the anterior (d, cross‐polarized light) and posterior (e, lambda cross‐polarized light) surfaces of the femur, showing variable thickness of the external lamellar zone. CCCB, compacted coarse cancellous bone; LB, lamellar bone. Scale bars as indicated.

The femur of NMMMH P‐48052 (Figure 6) is badly crushed, but its histological textures are well preserved. Only the distal end of the femur was preserved, and so the section was taken through the metaphysis rather than the diaphysis, which typically preserves the best record of growth. Accordingly, much of the growth signals recorded in the available sample might be anticipated to be more related to processes of cortical drift, growth cone expansion and active remodelling than they are to the primary growth of the individual. This appears to be the case: Nearly the entire cortex is formed of CCCB, capped in most areas by a thin veneer of lamellar bone at the periosteal surface (Figure 6d). In some areas, CCCB nearly reaches the periosteal surface (Figure 6e), indicating advanced modelling processes as areas that were formerly metaphyseal trabeculae have been transformed into cortical bone. On the anterior surface of the femur, a small wedge of fibrolamellar bone can be detected between the CCCB and the periosteal rind of lamellar bone, but no more than a few primary vascular canals are preserved within this area. The outer zone of lamellar bone preserves three bright lines on the anterior surface, but these cannot be traced to the posterior surface, where the CCCB nearly reaches the periosteal surface.

### 3.5 Tibia

**FIGURE 7:**
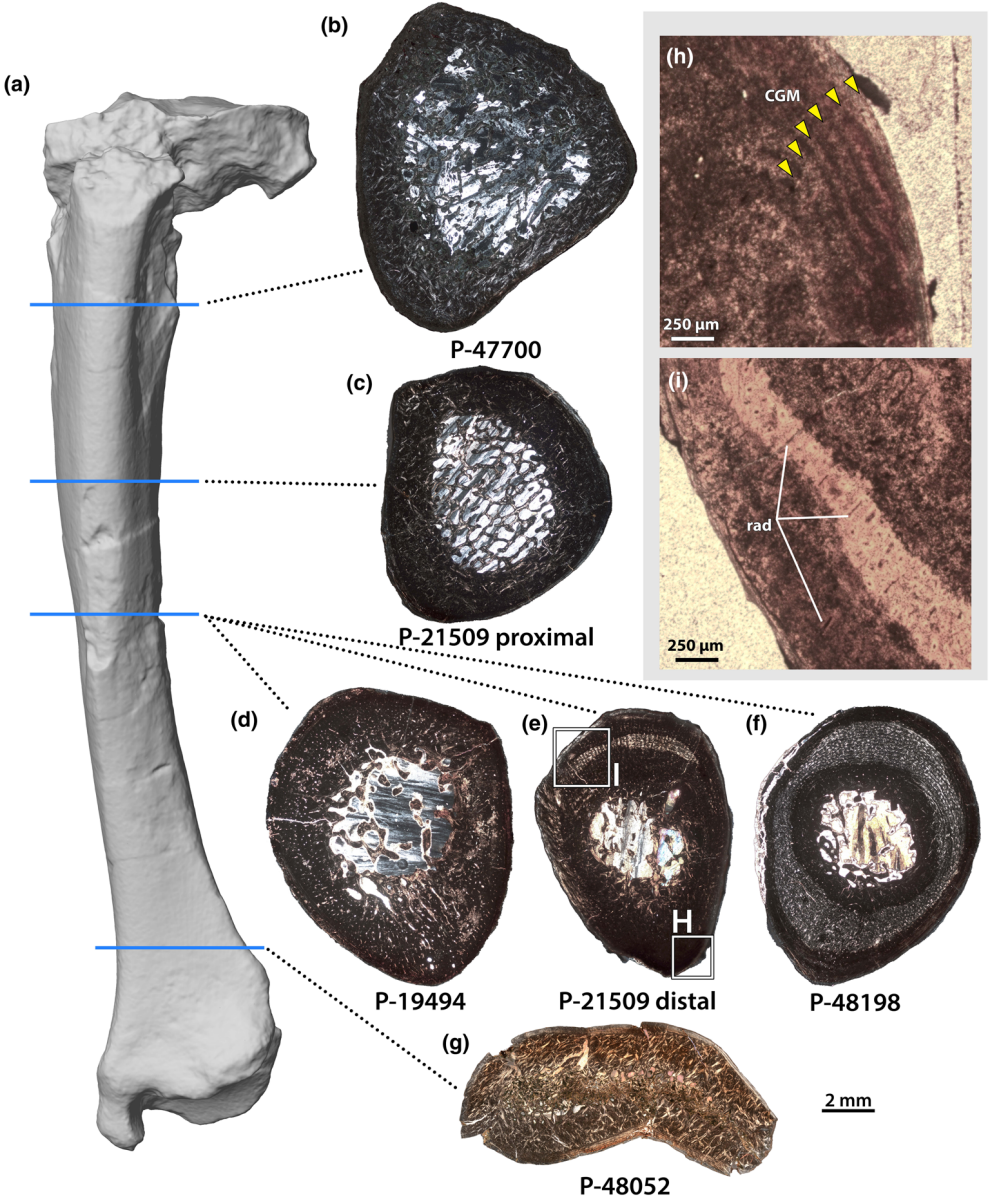
Tibiae of *Conoryctes comma*. Photogrammetric model of left tibia of NMMNH P‐48198 (a) in anterior view (distal towards the bottom), showing the approximate locations of thin sections of all specimens. From proximal to distal, these are NMMNH P‐47700 (b), NMMNH P‐21509 (c, e), NMMNH P‐19494 (d), NMMNH P‐48198 (f) and NMMNH P‐48052 (g). Close ups (h, i) of banding in the outer cortex (h) and short radial canals (i). Thin section images (b–g) to scale, anterior is down.

Tibiae were the most abundant element in our sample and were sampled from each of the five individuals other than NMMNH P‐79457 (Figure 7). However, these tibiae span a relatively narrow range of sizes (Table 2; 87–119 mm estimated lengths), and based on availability, sections had to be taken from a variety of positions along the shaft (Figure 7). Therefore, apparent variation in size, shape and internal architecture might not only strictly reflect ontogenetic differences between individuals but also processes of cortical drift and soft tissue attachment. From among the samples, sections of NMMNH P‐19494, P‐21509 and P‐48198 were taken closest to the minimum circumference of the shaft and are more likely to preserve an adequate record of body mass growth.

**TABLE. 2:** Comparative measurements of overlapping skeletal elements in the sample of *Conoryctes comma*.

Specimen number	Tibia transverse proximal width	Tibia transverse distal width	Tibia length	Femoral head transverse width
NMMNH P‐19494	—	19.62	103^a^	—
NMMNH P‐21509	21.45	—	87.57^a^	—
NMMNH P‐47700	29.06	—	119^a^	—
NMMNH P‐48052	—	20.39	107^a^	14.3
NMMNH P‐48198	27.68	21.52	113	—
NMMNH P‐79457	—	—	115^b^	15.4

*Note*: All measurements from Kynigopoulou et al. (2024).

^a^ Estimated from scaling based on NMMNH P‐48198.

^b^ Estimated by scaling femoral measurements to the estimated tibia size of NMMNH P‐48052 (i.e. double estimated).

Unfortunately, NMMNH P‐19494 (Figure 7d) is pervasively replaced with opaque minerals, which prevents detailed characterization of the tissue types. However, the vasculature of the inner cortex can be discerned as predominantly longitudinally oriented, and it was likely a fibrolamellar complex based on faint haloes around the vascular canals, which probably represent primary osteons. The medullary cavity is relatively open and well demarcated, and it is traversed by trabeculae. Through the cortex, there is a clear reduction of vascular density towards the periosteal surface, and this was presumably associated with a change to lamellar bone matrix as in other specimens. Three bright lines are visible in the external cortex.

Likewise, NMMNH P‐21509 (Figure 7c,e) exhibits considerable alteration of the tissues, but this varies along the length of the shaft. Whereas a sample proximally shows complete replacement (although overall architecture can still be determined), a section taken further distally closer to the midshaft has a crescent of unaltered tissue in the middle cortex (Figure 7e). The bone in this crescent is a fibrolamellar complex, with dense globular osteocytes in the woven portion and a low proportion of lamellar bone surrounding small vascular canals. The vascular canals are generally longitudinal, but this varies around the circumference of the bone, and an area on the medial side shows short radial canals oriented obliquely to the periosteal surface (Figure 7i). The bone matrix in this area also shows evidence of large collagen fibres extending parallel to the vasculature, and so these features are probably linked and likely related to a soft tissue attachment site. At least two growth marks are visible in the outer cortex around the entire circumference, but a region along the anteromedial side shows at least six alternating light and dark bands (Figure 7h). The more proximal section has well‐developed spongiosa filling the medullary cavity, which differs from sections at the midshaft but is similar to sections taken near the metaphyses (Figure 7). There appears to be a small ring of CCCB around the medullary cavity, and a thin inner cortex of what appears to be fibrolamellar bone, although discerning the bone matrices is difficult in this section. A transition to lamellar bone is evident close to the periosteal surface, and lamellae are clearly visible under cross‐polarized light.

**FIGURE 8:**
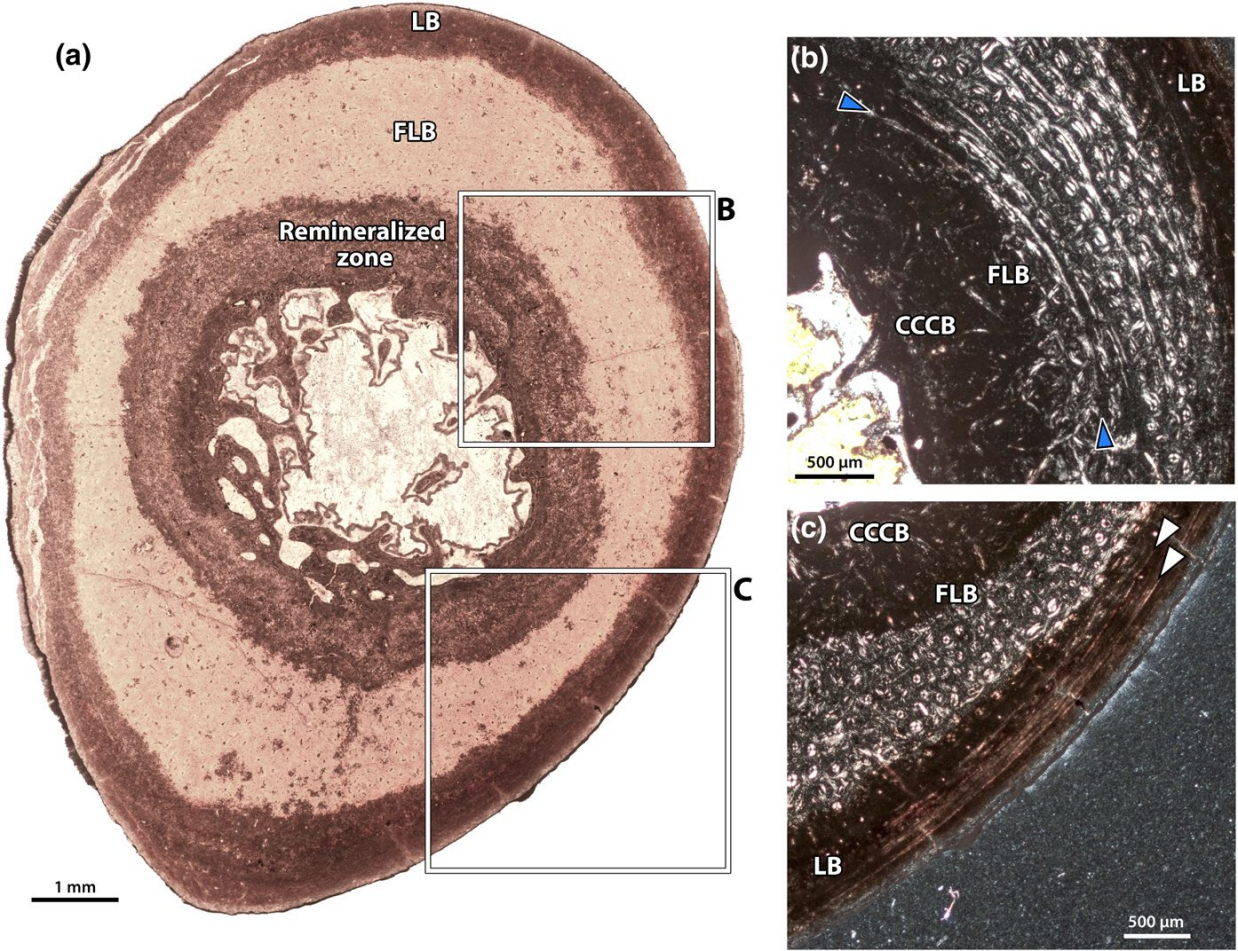
Tibia of NMMNH P‐48198. Thin section under normal light (a), showing partial remineralization of the cortex around the endosteal and periosteal surfaces, but intact palaeohistology in the intervening space. Close up (b) of internal annulus (curving between blue arrows) just exterior to the zone of compacted coarse cancellous bone, which likely reflects the weaning transition (see Discussion Section 4.2). Close up (c) of the external cortex, showing the transition between the primary vascular bone and external lamellar zone, with two bright lines interpreted as cyclical growth marks (arrows). CCCB, compacted coarse cancellous bone; FLB, fibrolamellar bone; LB, lamellar bone. Scale bars as indicated.

In NMMNH P‐48198 (Figures 7f and 8), two mineral replacement fronts, adjacent to the medullary cavity and the periosteal surface, have obscured some of the palaeohistological textures, but the middle of the cortex is unaltered (Figure 8a). The medullary cavity is small and relatively open, but with trabeculae extending partway into the lumen, and some small erosion cavities around its periphery. In some places, endosteal lamellae are developed, but there is only a very small zone of CCCB, roughly 400–500 μm wide, surrounding the medullary cavity (Figure 8a). The innermost cortical bone is mostly obscured by mineral replacement, but where it can be observed, it is composed of a fibrolamellar complex. Just towards the outside of the mineral replacement zone, there is a weakly expressed annulus in some regions (Figure 8b). Unfortunately, the continuity of the annulus and the characteristics of the tissues endosteal to it cannot be determined with certainty. External to the annulus, there is a single large zone of fibrolamellar bone with predominantly longitudinal vascularity (Figure 8). Osteocytes in this region are generally more lenticular and less dense than in the humerus of NMMNH P‐48052, but there are interspersed accumulations of globular osteocytes in the woven portions of the fibrolamellar matrix. Some of these are loci for opaque mineral replacement. The vasculature is slightly less dense than that of the humerus in other individuals (e.g. NMMNH P‐48052), but comparatively high relative to other bones in the sample. As in the other limb bones, there is a transition between the fibrolamellar inner cortex and a lamellar external cortex, marked by a prominent bright line (Figure 8c). Within the outer cortex, there are infrequent simple vascular canals, and a single cyclical growth mark that manifests as a bright line, for a total of two growth marks (Figure 8c).

The samples from NMMNH P‐47700 and P‐48052 were taken closer to the proximal and distal ends of the tibiae, respectively (Figure 7a,g). NMMNH P‐48052 is anteroposteriorly crushed, which has collapsed the medullary cavity, but it appears to have been similar to NMMNH P‐47700 in being filled by trabeculae. The cortices of both specimens are largely comprised of an extensive layer of CCCB, which is continuous with the medullary cavity and separated from the periosteal surface by a thin rind of lamellar bone. The zone of lamellar matrix varies in thickness around the cortex in both specimens, but in NMMNH P‐48052, the range of variation is greater, and the layer is thickest on the anterior and posterior margins of the bone, thinning transversely. Mineral replacement in NMMNH P‐47700 makes the detailed histological textures of the outer cortex difficult to discern, but in NMMNH P‐48052, numerous collagen fibres are visible extending obliquely to the periosteal surface, especially along the posterolateral and posteromedial margins. Growth marks are difficult to discern from lamellae in the external cortical zones of both specimens, but two accentuated lines are present in NMMNH P‐48052, whereas one prominent line is present in NMMNH P‐47700.

### 3.6 Fibula

The proximal end of the fibula of NMMNH P‐79457 was sampled. Like other bones of this individual, the primary tissues are largely altered by diagenetic minerals, but some textures can be discerned under cross‐polarized light. Most of the cortex is comprised of disorganized CCCB, which is continuous with the trabeculae of the medullary cavity. The tissues at the periosteal surface appear to be more organized than those internally based on consistent extinction patterns under cross‐polarized light, but their nature cannot be determined. No growth marks are apparent in the bone.

### 3.7 Manual/pedal elements

**FIGURE 9:**
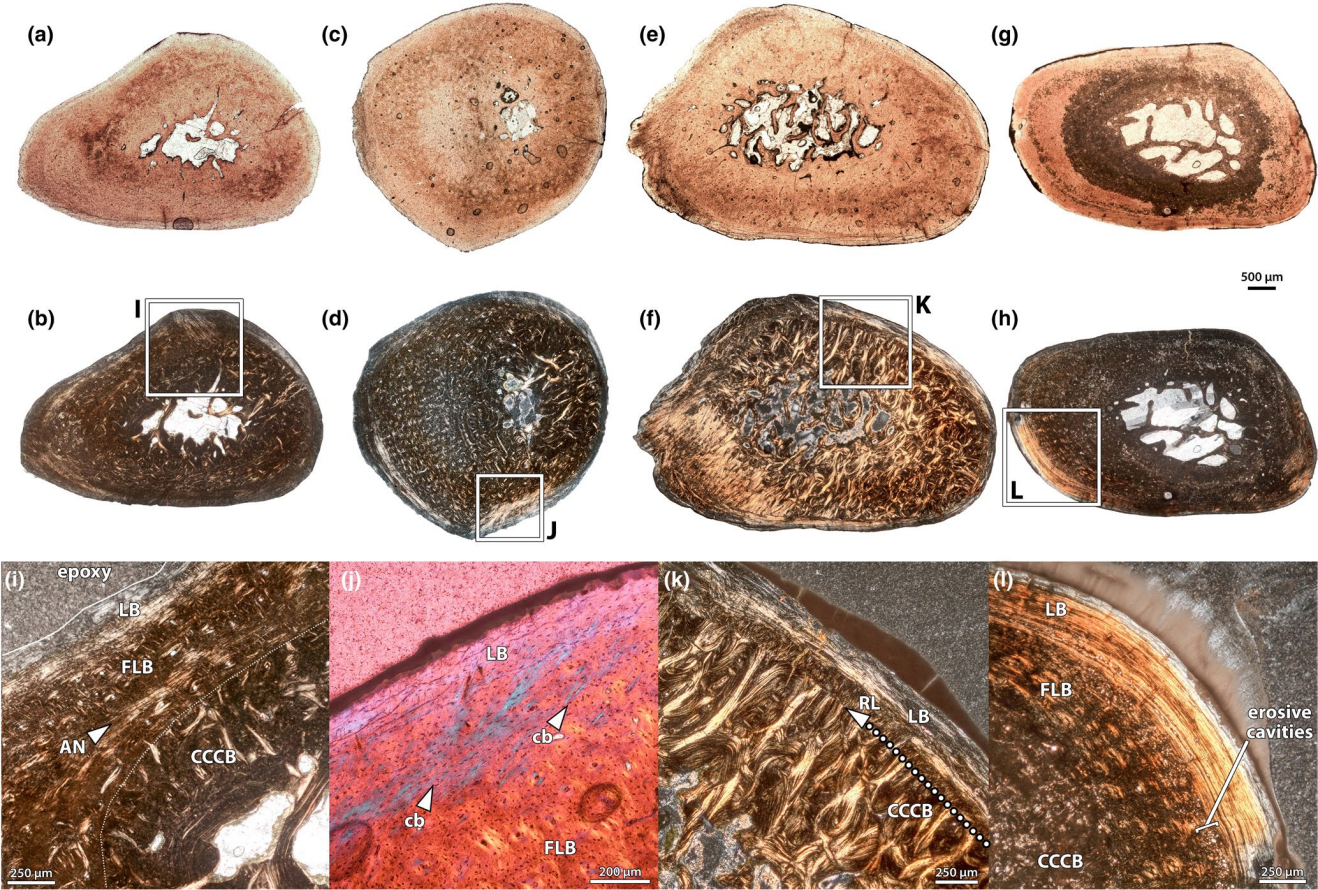
Small indeterminate manual/pedal bones and rib of NMMNH P‐48052. Indeterminate manual/pedal bones (a–f) and rib (g, h) under normal (a, c, e, g) and cross‐polarized (b, d, f, h) light, showing overall architecture of the elements. Close up (i) of internal annulus (arrow) just exterior to the zone of compacted coarse cancellous bone (CCCB) in (a). Close up (j) of collagen bundles (arrows) in the external lamellar zone (ELZ) of (c). Close up (k) of the transition between the CCCB and ELZ at a resorption line in the external cortex of (e). Close up (l) of the well‐developed ELZ of the rib, as well as erosive cavities at the transitions between tissue types that may be indicative of fungal erosion. AN, annulus; cb, collagen bundle; CCCB, compacted coarse cancellous bone; FLB, fibrolamellar bone; LB, lamellar bone; RL, resorption line. Scale bars as indicated.

Four small, rod‐like bones that probably represent elements of the hands and feet were sampled from NMMNH P‐48052 (Figure 9). Three of these elements have nearly identical osteohistological characteristics (Figure 9a,e,g), whereas the fourth (Figure 9c) shows different features. The three similar elements each have a central core of CCCB surrounding a small medullary cavity, which is spanned by trabeculae rather than having a sharp, resorbed margin. In two of these three elements, the CCCB is directly adjacent and separated by a tide line from an external zone of lamellar bone (Figure 9k), which in all three elements is relatively thicker (i.e. forms more of the cortex) than in the more proximal limb bones (Figure 9e,f,k). The third of the three similar bones, however, exhibits mintervening primary fibrolamellar tissue between the CCCB and the outer lamellar bone (Figure 9a,b,i). For the most part, this fibrolamellar bone is similar to that of most other *Conoryctes* bones sampled, with longitudinal vasculature and a low proportion of woven bone. A notable feature, however, is the presence of a ring of parallel‐fibred to lamellar bone in the inner cortex (Figure 9i), close to (and in some places truncated by) the transition with the CCCB. This internal feature is especially similar to the annulus in the internal cortex of the tibia of NMMNH P‐48198 (Figure 8b), in both its position in the inner cortex close to the CCCB zone and its variable expression around the circumference of the cortex.

The fourth element sampled (Figure 9c,d,j) shows a different cross‐sectional shape from the others, more circular than triangular or elliptical. Unlike the other elements, the medullary cavity of this bone is smaller, off‐centre relative to the periosteal surface, and there is a much smaller region of CCCB surrounding it. Accordingly, the majority of the cortex is comprised of primary fibrolamellar bone. Like in other specimens, the inner cortex has longitudinal vasculature, but there is a higher proportion of woven‐fibred bone than in other samples, particularly in the regions of the cortex along the flatter sides of the element (presumably medial and lateral). Some linear features are visible close to the border between the CCCB and the inner cortex (Figure 9c), but they do not extend long distances and they are not distinct under cross‐polarized light. There is an external zone of lamellar bone, which is demarcated from the fibrolamellar bone of the inner cortex by a faintly expressed LAG. At least one other accentuated line is present within the outer lamellar zone, although it is unclear whether it is a cyclical growth mark. Collagen bundles are well developed near the periosteal surface of this element (Figure 9j).

### 3.8 Ribs

Two ribs of NMMNH P‐48052 were sectioned just distal to the capitulum (Figure 9g,h,l). Their osteohistological characteristics are similar to each other and to the other bones in the sample. The medullary cavities are spanned by trabeculae and surrounded by relatively small regions of CCCB. External to this, the majority of the cortex is composed of a fibrolamellar complex with longitudinal vascularity organized into circumferential rows (Figure 9l). Numerous collagen fibres are present throughout this region, but some patches are oriented parallel to the periosteal surface, giving the superficial appearance of parallel‐fibred bone matrix. However, osteocytes in these areas remain globular, rather than lenticular as would be expected in parallel‐fibred bone matrix, and so these areas are better interpreted as collagen fibres arising from entheses. Like other bones, towards the periosteal surface the fibrolamellar complex transitions to an outer cortex of lamellar bone at a prominent LAG. The bone of the outer cortex is mostly avascular, although there are several small cavities in one area just external to the LAG, and similar small cavities are present elsewhere throughout the specimens (Figure 9l). These appear to be erosive and likely result from deterioration or decomposition of the specimen before or during burial (possibly by fungi), but their consistent presence in the same locations in two separate bones might also suggest that they correspond to some biological feature that predisposed the area to degradation. The lamellae within the lamellar bone of the outer cortex are well developed (Figure 9l) and there are at least two prominent lines within this zone. However, like other specimens with well‐developed lamellae, distinguishing these lamellar lines from LAGs is difficult. Altogether, this zone can be classified as an outer circumferential layer (external fundamental system) (Woodward et al., 2011).

### 3.9 Indeterminate bone fragments

A spate of indeterminate bone fragments (*n* = 5) was sampled from P‐47700 and P‐48052. The identities of these fragments within the skeleton could not be ascertained, but each can be confidently associated with the individual because they were collected together, are identical in taphonomic and diagenetic conditions and are within the size range of other elements.

The fragments from NMMNH P‐47700 add little information on the overall growth of this individual, as they are consistent with the histological signals from other bones. Two fragments have large medullary cavities with extensive CCCB and only a thin outer cortex of lamellar bone with no intervening fibrolamellar zone. The third fragment is pervasively replaced with opaque minerals and its histological characteristics cannot be determined.

**FIGURE 10:**
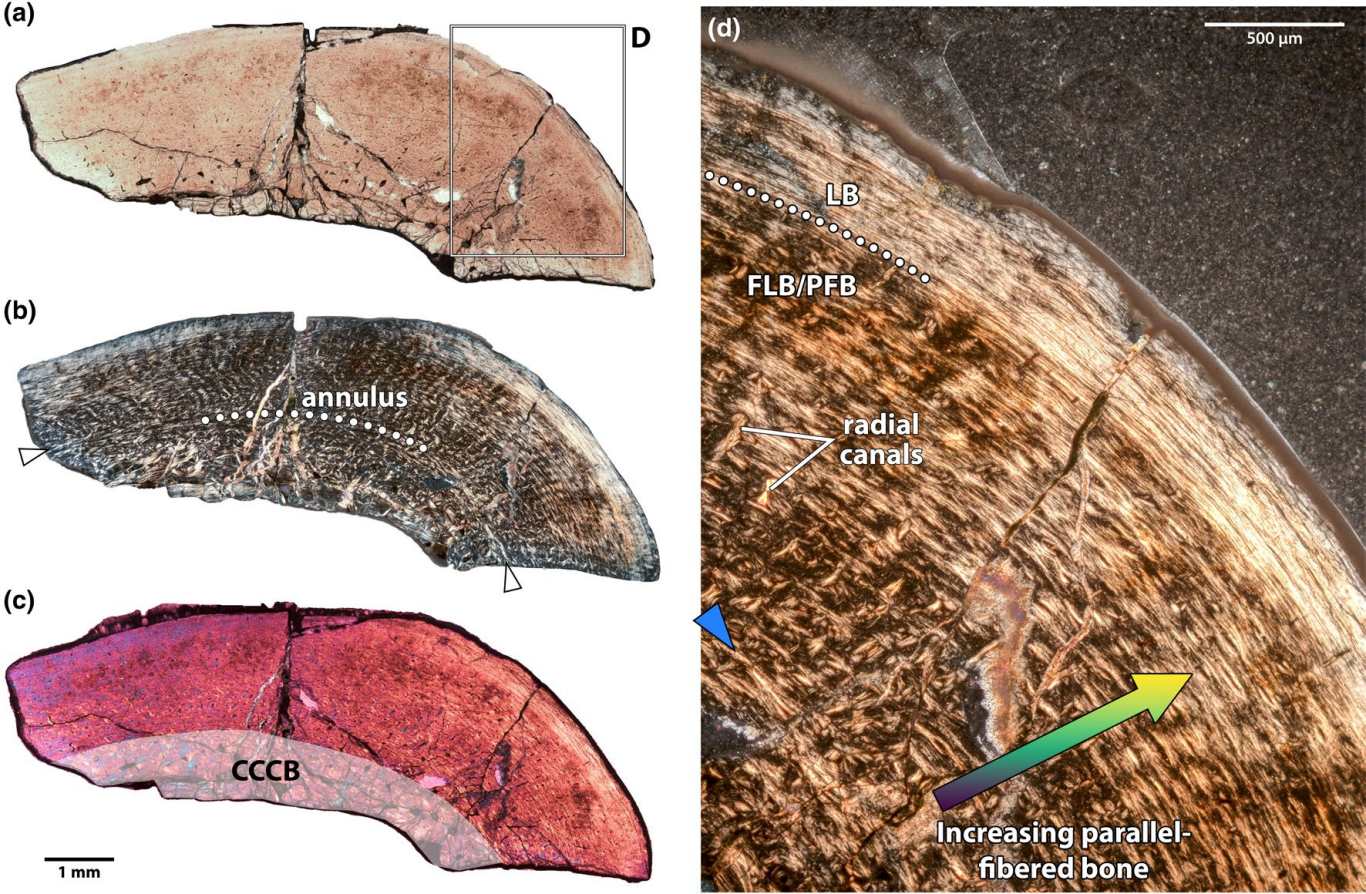
Indeterminate bone midshaft fragment of NMMNH P‐48052. Overview of thin section under normal (a), cross‐polarized (b) and lambda cross‐polarized (c) views, showing overall architecture, presence of faint internal annulus (white arrows and dotted line) and extent of CCCB in the inner cortex (shaded area). Close up (d) of external cortex, showing increased component of parallel‐fibred matrix periosteally within the primary vascular bone, and the transition to the external lamellar zone (dotted line). Examples of radial canals, more prevalent in this part of the bone, are indicated. Blue arrow indicates faint internal annulus. CCCB, compacted coarse cancellous bone; FLB/PFB, zone of fibrolamellar and parallel‐fibred bone; LB, lamellar bone. Scale bars as indicated.

A flake of bone from NMMNH P‐48052 has more information to offer (Figure 10). There is no diagenetic alteration and so the histological textures are clearly visible. The fragment includes both the endosteal and periosteal surfaces, and thus provides a corroborating record of growth for the limb bones of this individual, which in some cases had to be sampled far from the minimum circumference of the diaphysis. The endosteal surface of the flake is relatively flat and clean, without the trabecular protrusions that characterize other limb bones in the sample. Nevertheless, several invaginations are present, and there is continuity between the medullary cavity and some of the large erosive cavities that line the inner third of the cortex. The extent of these erosive cavities demarcates the internal zone of CCCB (Figure 10c), which grades conformably into the fibrolamellar complex that makes up most of the cortex. There is a higher proportion of woven matrix in the fibrolamellar bone in this flake than in most of the other bones of this individual, but there are marked changes in bone matrix through the cortex. Whereas woven bone is abundant within the fibrolamellar complex endosteally, this grades into a zone of parallel‐fibred bone periosteally, with lenticular osteocytes, before the transition to lamellar bone in the outer cortex (Figure 10d). Thus, in some areas, about half of the central, vascularized zone of the cortex is comprised of parallel‐fibred bone rather than fibrolamellar bone. Like other limb bones, vasculature in the inner cortex is primarily longitudinal, but it is slightly more dense in this element than others, and there is variation around the cortex and along the endosteal–periosteal axis. Towards the more curved end of the flake, the number of radial canals increases (Figure 10d), whereas towards the flatter end, vasculature is more reticular and longitudinal. Within the inner cortex, the vasculature is more reticular endosteally, and more longitudinal periosteally, with the transition occurring in the same location as the fibrolamellar–parallel‐fibred matrix transition described above. No clear growth marks are present in the inner cortex, although as in the indeterminate pedal bone of the same individual and in the tibia of NMMNH P‐48198, there is a faint annulus just external to the zone of CCCB (Figure 10b,d). The transition between the inner and outer cortex is more gradual in this flake of bone than in other bones of this individual, in part because of the change to parallel‐fibred bone partway through the inner cortex (Figure 10d). The LAG that sharply separates these regions in other elements is only weakly expressed in this flake, and not consistently around the entire cortex. There are no clear LAGs in the outer cortex, but two faint bright lines are present under normal light, probably representing cyclical growth marks.

### 3.10 Tooth root

**FIGURE 11:**
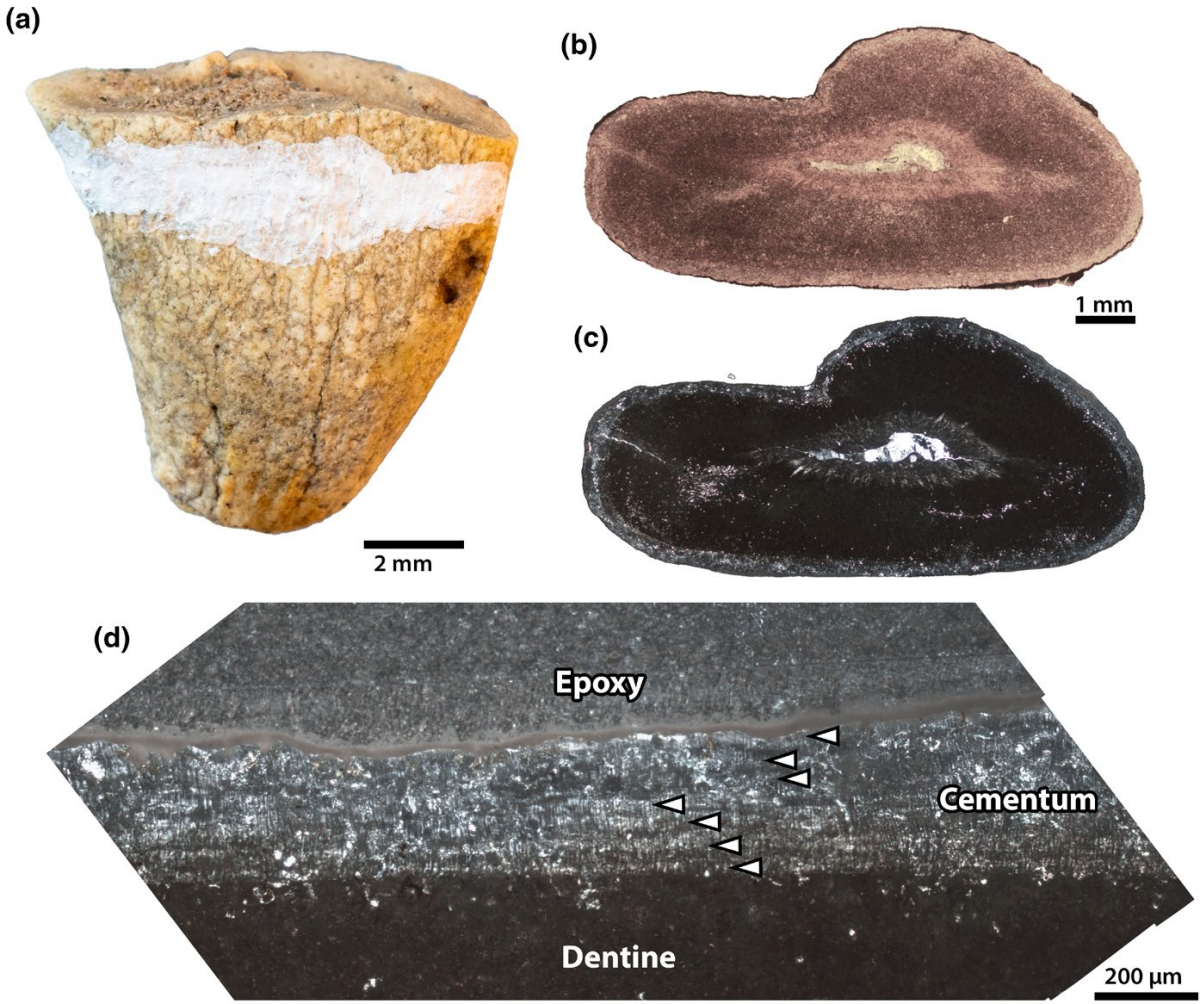
Dental palaeohistology of *Conoryctes comma*. Partial tooth root of indeterminate molar associated with NMMNH P‐21509 (a), showing plane of section (white mark). Overview of thin section (b, c) under normal (b) and cross‐polarized (c) light, showing extensive remineralization and destruction of histological features, except in the bright ring of cementum surrounding the root. Close up (d) of cementum, showing tentative banding of bright and dark lines. Seven bright lines are indicated by white arrows. Scale bars as indicated.

A root of an indeterminate tooth of NMMNH P‐21509 was sectioned transversely as close to the crown as possible (Figure 11). Extensive diagenetic alteration obscures nearly all incremental growth features of the tooth under normal light, but the cementum is clearly distinguishable from the dentine. Under cross‐polarized light, some faint banding is apparent in the cementum. At least three clear pairs of alternating light and dark bands are discernible in the cementum nearest the dentine, but banding becomes less clear towards the external cementum surface. Nevertheless, rotating the slide under cross‐polarized light to alter the extinction patterns, we were able to identify seven total bright bands of consistent thickness (Figure 11d).

## 4 DISCUSSION

### 4.1 Growth patterns in *C. comma*

Overall, the growth patterns recorded in the limb bones of our sample are consistent both between elements of a single skeleton, and between the different individuals preserved. All specimens exhibit a transition from more rapidly growing fibrolamellar or parallel‐fibred bone internally to an outer cortex of slow‐growing lamellar bone (e.g. Figure 5d), indicating that each individual had decreased in growth rate can be considered skeletally mature. This is consistent with the relatively similar estimated sizes of the bones (Table 2) and the fusion of epiphyseal plates where preserved. Endosteally, many specimens exhibit zones of CCCB of variable sizes (e.g. Figure 9k). Where these are present in samples near the metaphyses of the elements, they probably reflect metaphyseal growth dynamics as described by Enlow (1962, 1963). In these regions, they are relatively large, comprising a greater proportion of the cortex. However, CCCB is also present in some of our samples in small zones close to the midshaft, where it would normally be removed through ontogeny by expansion of the medullary cavity (Enlow, 1962, 1963). As discussed below, the nature of this CCCB is unclear, but it may reflect cortical thickening through ontogeny instead of metaphyseal growth dynamics (Montoya‐Sanhueza & Chinsamy, 2017), potentially related to fossorial adaptations. Where primary bone is visible in the inner cortex between the outer cortex and endosteal CCCB, it is a fibrolamellar complex with woven‐fibred or less frequently parallel‐fibred bone (e.g. Figures 3 and 10). Growth marks are difficult to discern in many elements because of diagenetic remineralization, but where they are found, they are consistent in position and number between elements of the same skeleton. Excluding the annulus present in some elements (see below), cyclical bone growth marks are consistently positioned only within the outer lamellar cortex, rather than in the inner fibrolamellar cortex, suggesting that the fibrolamellar zone was deposited in a single year or less (Castanet, 2006; Castanet et al., 2004; Köhler et al., 2012). This further suggests that *C. comma* reached sexual maturity within 1 year, a transition which is usually accompanied by decreased growth rate (Calderón et al., 2019; Lee & Werning, 2008). Altogether, based on our sample, it appears that the growth patterns preserved in each element generally reflect the overall growth of the individual, with predictable variation arising from sample position along the shaft, rather than the peculiarities of any particular region of the skeleton. However, the diagenetic alteration of the tissues may obscure some growth marks.

Based on cyclical growth marks, which we interpret as annual (Castanet, 2006; Castanet et al., 2004; Köhler et al., 2012), most individuals in the sample were 3–5 years old at death (Table 3). However, there is a possible discrepancy between the number of annual marks identified in the bones and the cementum of one individual (NMMNH P‐21509). In this individual, seven cementum bands are tentatively identified in the root (Figure 11d), but this tooth is poorly preserved and these bands cannot be traced for long distances. The tibia of this individual shows at least two easily discernible growth marks, and in some areas, there is banding that may indicate the presence of up to six growth marks (Figure 7h). This bone is likewise poorly histologically preserved, and so it is difficult to be confident of the number of growth marks or to trace these around the cortex. This discrepancy may simply be the result of poor preservation, or it could indicate that osteogenesis ceased partway through life, resulting in fewer bone growth marks (but not cementum annulations) than years lived, as occurs in some mammals today (Castanet et al., 2004). Discerning between these options is difficult with the available material. Nevertheless, based on the slightly better preservation of the cementum, bone banding that possibly indicates additional growth marks in the tibia, and the fact that bone growth marks can underestimate age in old individuals (Castanet et al., 2004), we interpret this individual to be 7 years of age, making it the oldest in our sample. Thus, in our sample, ages at death range from 3 to 7 years (Table 3).

**TABLE 3:** Palaeohistological observations in *Conoryctes comma*.

Specimen number	Element	Tissues observed (internal to external)	Number of cyclical growth marks	Degree of diagenetic alteration
NMMNH P‐19494	Tibia	CCCB → FLB → LB	4	+
	Limb fragment	CCCB → FLB → LB	4	+
NMMNH P‐21509	Tooth root	Dentine → Acellular Cementum	7	+
	Tibia	CCCB → FLB → LB	6?	+
NMMNH P‐47700	Humerus	CCCB → FLB → LB	2	~
	Tibia	CCCB → LB	2	+
	Limb fragment	CCCB → LB	−	~
	Bone fragments	CCCB	−	+
NMMNH P‐48052	Humerus	CCCB → FLB → LB	3	0
	Ulna	CCCB → FLB → LB	4?	~
	Femur	CCCB → LB	3	−
	Tibia	CCCB → LB	4	−
	Limb fragment	CCCB → FLB → An → FLB → LB	3?	0
	Autopodial bone	CCCB → LB	3	0
	Ribs	CCCB → FLB → LB	3	~
	Bone fragments	CCCB → FLB → An → FLB → LB	3	0, −
NMMNH P‐48198	Tibia	CCCB → FLB → An → FLB → LB	3	~
NMMNH P‐79457	Humerus	CCCB → FLB → LB	1	+
	Fibula	CCCB	−	+
	Radius	EL → CCCB → FLB → LB	4	+

*Note*: An, annulus; CCCB, compacted coarse cancellous bone; EL, endosteal lamellae; FLB, fibrolamellar complex; LB, lamellar bone; +, high or complete; ~, partial; −, low; 0, pristine.

### 4.2 Potential weaning mark

We identify an annulus in some bones in the innermost cortex (Figures 8, 9, 10), indicating a brief decrease in growth rate. Whereas this could be a cyclical growth mark reflecting an annual hiatus in growth, this seems unlikely for several reasons. First, it differs from other growth marks in the sample, which manifest as lines of arrested growth rather than annuli. Second, it is only present in some individuals, even where multiple individuals were sampled at the same position (e.g. the midshaft tibiae of NMMNH P‐19494, P‐21509 and P‐48198). Third, this mark is only present in some elements of an individual, and in those elements, it is better developed in some areas compared to others. Finally, the annulus is positioned very close to the medullary cavity, which suggests that it reflects an event early in life at a small body size (see more below), which would be unexpected of an annual mark for a eutherian mammal. Although its position does not exclude an annual growth mark, most eutherian mammals (excepting some primates) exhibit initially rapid postnatal growth because of parental care (Case, 1978; Gaillard et al., 1997), an innovation seemingly inherited from relatively ancient origins (Newham et al., 2024). Indeed, many mammals further synchronize their oestrous cycles to annual cycles to optimize seasonal emergence of young and reproductive timing, avoiding births shortly before the unfavourable season (Smith, 2024). Instead, we consider a non‐cyclical origin more likely, and two early life‐history events seem particularly salient: birth and weaning.

**FIGURE 12:**
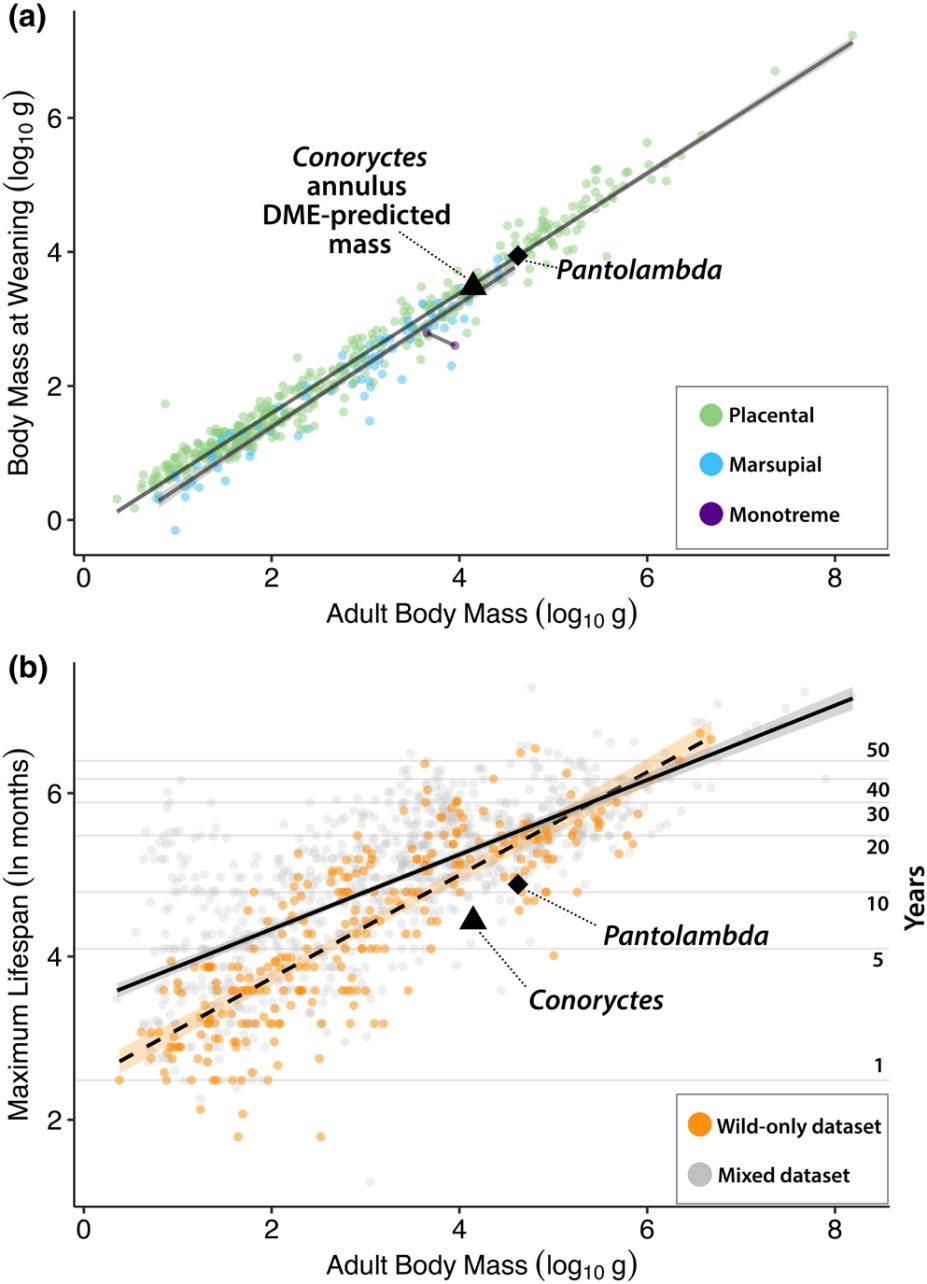
Life history estimates of *Conoryctes comma* compared to extant mammals. Scatterplots of adult body mass (log_10_ g) compared to mass at weaning (a; log_10_ g), and maximum lifespan (b; ln months), based on data from the PanTheria life‐history dataset (Jones et al., 2009) and the Newham et al. (2020) lifespan dataset. The annulus in *Conoryctes* (triangle) and a similar weaning mark in *Pantolambda* (diamond) closely match the tight relationship in therian mammals. Both species appear to have shorter maximum lifespans based on fossil data, likely a consequence of sampling. Horizontal lines in (b) show untransformed values (right axis label).

Osteological birth lines are well documented in ontogenetic series of eutherian mammals and other vertebrates, distinguished by both the presence of a distinct boundary and by relative growth rates recorded in the tissues on either side of the boundary. In mammals, tissue characteristics usually record an increase in growth rate across this transition (Garrone et al., 2019; Nacarino‐Meneses & Köhler, 2018), reflected by a decrease in the proportion of parallel‐fibred matrix and an increase in the area of vascular canals. In our *Conoryctes* sample, we do not observe either of these changes, and instead the features we do observe (an annulus and decreases in osteocyte density and size) would tend to indicate a decrease in growth rate, rather than an increase. It is also noteworthy that the annulus corresponds to a much larger percentage of adult body size than expected for a neonate, based on developmental mass extrapolation. Whereas the mark occurs at ~35% of adult tibia circumference, or 21% (~2900 g) of adult body mass, regression of neonate mass to adult body size in living eutherians (*n* = 1016; *p* = 0.00; *y* = 0.88*x* − 0.87; *R* ^2^ = 0.939) suggests that *Conoryctes* might be expected to give birth to neonates approximately 600 g in mass, roughly five times smaller than the estimated mass.

Instead, the growth mark bears more similarity to weaning marks described in the literature, in its manifestation as an annulus and its correspondence to a decrease in growth rate (Calderón et al., 2021; Castanet et al., 2004; Funston et al., 2022; Köhler et al., 2023; Morris, 1970). Indeed, the position of the mark corresponds almost exactly to the estimated weaning mass of *Conoryctes*, based on the tight correlations of adult body mass with weaning mass (Figure 12) in not only eutherians (*n* = 392; *p* = 0.00; *y* = 0.894*x* − 0.192; *R* ^2^ = 0.98), but also therian mammals more broadly (*n* = 484; *p* = 0.00; *y* = 0.895*x* − 0.232; *R* ^2^ = 0.97). These regressions predict values of 3283 and 3018 g, respectively, much closer to the mass estimated from the growth mark (2900 g). This result strongly suggests that this mark occurred at the typical weaning mass for an animal of this size. Notably, on the basis of dental trace elements, another ‘archaic’ Palaeocene eutherian mammal from the San Juan Basin of New Mexico, *Pantolambda*, weaned after 31–56 days at the same percentage (21%) of adult body mass (Funston et al., 2022), which supports the notion that the relationship between weaning mass and adult mass was already established in closely related animals. Critically, the postcranial bones of some *Pantolambda* individuals exhibit nearly identical marks to those in *Conoryctes*, but which can be more confidently identified as weaning marks on the basis of dental trace elements reflecting breastfeeding (Funston et al., 2022).

Thus, although our data tentatively support the interpretation of this feature as a weaning mark, they highlight the difficulty of identifying weaning in extinct mammals, and the value of extant validation studies to further characterize non‐cyclical growth marks (Calderón et al., 2021; Köhler et al., 2023; Nacarino‐Meneses & Köhler, 2018). Ideally, to be confident in the interpretation of this mark in *Conoryctes*, it would be compared to an ontogenetic framework describing typical osteohistological changes across the weaning transition and the frequency with which this transition appears as a discrete mark. With the exception of Calderón et al.'s study (Calderón et al., 2021), descriptions of weaning marks in bones have been opportunistic, rather than part of systematic studies to identify and characterize them.

### 4.3 Functional implications

Life‐history traits like sexual maturity, growth rate and longevity have often been inferred from osteohistology, but other aspects like function have traditionally been less straightforward to infer from osteohistology alone. Recent work, however, has recognized several relatively robust osteohistological correlates for fossoriality (Amson et al., 2022; Bhat et al., 2019; Legendre & Botha‐Brink, 2018; Montoya‐Sanhueza & Chinsamy, 2017; Nakai & Yokohata, 2024; Straehl et al., 2013), which are relevant in light of interpretations of fossorial adaptations in *Conoryctes* (Kynigopoulou et al., 2024).

Bone compactness has been demonstrated to correspond to substrate preference, and several studies show that fossorial (particularly subterranean) animals have enhanced bone compactness relative to other mammals (Amson et al., 2022; Bhat et al., 2019; Legendre & Botha‐Brink, 2018; Montoya‐Sanhueza & Chinsamy, 2017; Straehl et al., 2013). In *Conoryctes*, the limb bones are generally robust, but the forelimb bones in particular have enhanced compactness in cross section relative to the hindlimb bones, resulting from small medullary cavities frequently infilled with trabeculae (Figures 2, 3, 4, 5). From a cursory survey based on available thin sections (G. Funston, unpubl. data), the forelimb bones of *C. comma* do indeed show greater compactness than other early Palaeocene mammals (Table 4). However, these data have been selected based on availability, and the initial sample was chosen to represent as broad a taxonomic range as possible, and so they may not capture the full picture of bone compactness in the early Palaeocene (e.g. see Shelley et al., 2021).

**TABLE 4:** Comparison of bone compactness based on thin sections from Palaeocene mammals.

Taxon	Specimen (NMMNH)	Humerus	Radius	Ulna
*Conoryctes*	P‐48052	+		+
	P‐47700	+		
	P‐74975		+	
*Acmeodon*	P‐54499		−	
*Goniacodon*	P‐47910	−		
*Loxolophus*	P‐36633	−		—
*Mithrandir*	P‐3083	—	=	
*Pantolambda*	P‐27844^a^	−	−	—
*Periptychus*	P‐35194	−	−	=
*Prodiacodon*	P‐60135	—		—
*Tetraclaenodon*	P‐51388	−		

*Note*: +, high bone compactness; −, lower bone compactness than *Conoryctes*; —, much lower bone compactness than *Conoryctes*; =, comparable bone compactness to *Conoryctes*. All data from Edinburgh Fossil Thin Section Collection (GFF, unpubl. data).

^a^ Juvenile specimen.

Nevertheless, in addition to overall greater bone compactness than its contemporaries, the finer osteohistological details of the forelimbs in *Conoryctes* are also consistent with fossoriality. Numerous studies have drawn a link between CCCB and progressive cortical thickening in fossorial taxa (Amson et al., 2022; Bhat et al., 2019; Legendre & Botha‐Brink, 2018; Montoya‐Sanhueza & Chinsamy, 2017, 2018; Straehl et al., 2013). For example, Montoya‐Sanhueza and Chinsamy (2017) comprehensively detailed ontogenetic change in limb bone compactness in the molerat *Bathyergus suillus*, providing an ideal comparison for *C. comma*. The ontogenetic patterns in *Conoryctes* appear to mirror those in *Bathyergus*, where they reflect progressive cortical thickening through life that results in lower resorption rates at the endosteal surface, paired with increased deposition, resulting in extensive zones of CCCB. The presence of extensive zones of CCCB at the midshafts of the forelimb bones in *Conoryctes* suggests a similar mechanism was at play. The consistency of these palaeohistological signals with morphological signals (Kynigopoulou et al., 2024) supports the idea that *Conoryctes*, and likely other taeniodonts, were specialized for fossorial behaviour.

### 4.4 Life history and the end‐Cretaceous extinction

The growth trajectory of *Conoryctes*, based on its osteohistology, was characterized by rapid growth to near‐maximum body size, followed by several years of slower growth. This pattern, and the tissues which attest to it, are more consistent with the growth strategies observed in living eutherians (i.e. placentals) than those of metatherians (i.e. marsupials) (Case, 1978; Chinsamy & Hurum, 2006; Enlow & Brown, 1958; Weaver et al., 2022; Werning, 2013). Several recent studies show that marsupials tend to exhibit slower post‐weaning growth rates, and therefore have more parallel‐fibred and lamellar bone throughout the cortex, with woven bone deposited only early in life during the weaning period (Chinsamy & Hurum, 2006; Chinsamy & Warburton, 2021; Walker et al., 2020; Werning, 2013). Because of this slow post‐weaning growth, marsupials tend to take multiple years to get to adult body size, depositing multiple growth marks within predominantly parallel‐fibred or lamellar cortices. Some large marsupials (>50 kg) do exhibit a greater proportion of woven‐fibred bone matrix (Chinsamy & Warburton, 2021), but because they take multiple years to reach maturity, these zones are still interspersed with multiple growth marks, and they occupy a lower proportion of the cortex than in eutherians. *Conoryctes*, in contrast to known extant and extinct marsupials (Chinsamy & Warburton, 2021; Walker et al., 2020; Werning, 2013), has cortices with extensive fibrolamellar bone and a high proportion of woven matrix for nearly its entire growth, covered in only a thin ring of lamellar bone formed towards the end of life (e.g. Figures 2 and 8, 9, 10). This indicates a far more placental‐like than marsupial‐like growth strategy, despite the likely position of *Conoryctes* as a stem placental outside the crown (Fox, 2016; Halliday et al., 2017; Rook & Hunter, 2013; Shelley and Paleocene Mammal Working Group, 2022).

Overall, the growth dynamics recorded by the bones in *Conoryctes* compare closely with the life history estimated for *Pantolambda* (Funston et al., 2022). Both taxa appear to have reached sexual maturity in about 1 year, which is typical of many small‐ to medium‐bodied extant mammals (Smith, 2024). However, each also appears to have had a shorter lifespan (Figure 12) than comparably sized mammals today (Funston et al., 2022; Jones et al., 2009; Newham et al., 2020); in the case of *Pantolambda*, less than half the expected lifespan. Of course, it is difficult to accurately estimate lifespan in fossil populations, as few individuals reach maximum ages in extant populations and even fewer of these would have been adequately preserved to estimate age. Therefore, it is highly probable that the maximum lifespans for any extinct mammal will be underestimated.

The finding of an apparently placental‐like rapid early‐life growth rate in *Conoryctes* may have implications for the life histories of Mesozoic eutherians and their roles in the end‐Cretaceous extinction. Taeniodonta are one of the few Palaeocene lineages with possible representatives or very close relatives in the Cretaceous: The relatively large‐bodied *Schowalteria* and a number of ambiguous cimolestan antecedents, whose relationships remain broadly unresolved (Archibald & Averianov, 2012; Lopatin & Averianov, 2023; O'Leary et al., 2013; Rook & Hunter, 2013; Velazco et al., 2022; Wible et al., 2009). The evidence for placental‐like early life growth strategies in both Taeniodonta and Pantodonta hints that their distinctive precocial development was common within non‐placental eutherians and may have been present in the Cretaceous *Schowalteria*. This would support the notion that at least some aspects of placental‐like precociality were established in stem eutherians, and thus that this life history may have helped them increase in size relatively rapidly after the extinction event (Funston et al., 2022). However, shared growth dynamics between *Conoryctes*, *Pantolambda* and some multituberculates (Weaver et al., 2022) suggest that eutherian mammals were not unique in these characteristics, and that other factors were likely also at play in the Palaeocene rise of placentals. Further work sampling non‐placental eutherians and non‐therian mammals will help to better constrain the origin of precociality in mammals, and its impact on their evolution and diversification.

## AUTHOR CONTRIBUTIONS

GFF conceived of the study, created and analysed the thin sections, wrote the manuscript and created the figures. ZK identified the material, assisted in study design, provided data and edited the manuscript. TEW collected the specimens, assisted in study design, provided data and edited the manuscript. SLB conceived of the study, supervised the research and edited the manuscript.

## CONFLICT OF INTEREST STATEMENT

The authors declare that they have no conflicts of interest.

## Data Availability

Images of thin sections used in the study are available at https://doi.org/10.5061/dryad.vhhmgqp6f.
